# Dissecting diffuse large B‐cell lymphoma heterogeneity: New insights from a state‐specific molecular characterisation

**DOI:** 10.1002/ctm2.70757

**Published:** 2026-08-19

**Authors:** Robel Papotti, Filippo Vit, Tamara Bittolo, Federico Pozzo, Stefania Bettelli, Samantha Pozzi, Luca Braglia, Elisa Forti, Arianna Di Napoli, M. Christina Cox, Tamar Tadmor, Pellegrino Musto, Leonardo Flenghi, Martina Quintini, Giovanna R. Mansueto, Sara Galimberti, Valentina Donati, Michele Spina, Alberto Zamò, Andreas Rosenwald, Riccardo Bomben, Stefano Sacchi, Valter Gattei

**Affiliations:** ^1^ Clinical and Experimental Onco‐Hematology Unit Centro di Riferimento Oncologico di Aviano (CRO), I.R.C.C.S. Aviano Italy; ^2^ Molecular Pathology and Predictive Medicine Unit Modena University Hospital Modena Italy; ^3^ Oncohematology and Osteoncology Unit Modena University Hospital Modena Italy; ^4^ Pathology Unit Sapienza University Sant'Andrea Hospital Rome Italy; ^5^ Fondazione Policlinico Tor Vergata University Hospital of Rome Rome Italy; ^6^ Hematology Unit Bnai Zion Medical Center Haifa Israel; ^7^ Aldo Moro University School of Medicine and Unit of Hematology and Stem Cell Transplantation AOU Consorziale Policlinico Bari Italy; ^8^ Department of Emergency and Organ Transplantation Azienda Ospedaliera di Perugia Perugia Italy; ^9^ Pathology Unit IRCCS‐CROB of Rionero in Vulture Vulture Italy; ^10^ Hematology Unit Azienda Ospedaliero‐Universitaria Pisana Pisa Italy; ^11^ Pathology Unit Azienda Ospedaliero‐Universitaria Pisana Pisa Italy; ^12^ Oncologia Medica e dei Tumori Immuno‐Correlati Centro di Riferimento Oncologico di Aviano (CRO), I.R.C.C.S. Aviano Italy; ^13^ Pathology Unit Julius‐Maximilians‐Universität Würzburg Germany

**Keywords:** digital deconvolution, DLBCL, lymphoma, tumour microenvironment, EcoTyper

## Abstract

**Background:**

Diffuse large B‐cell lymphoma (DLBCL) is a biologically and clinically heterogeneous disease.

**Methods:**

In this study, we analysed a real‐world cohort of 178 newly diagnosed DLBCL patients homogeneously treated with R‐CHOP‐like regimens, integrating RNA sequencing, targeted mutational profiling and digital deconvolution using the EcoTyper algorithm.

**Results:**

Five malignant B‐cell states (S1–S5) were identified, each reflecting distinct differentiation and transcriptional programs with prognostic relevance, S1 and S5 representing the most favourable and adverse subtypes, respectively. EcoTyper‐defined lymphoma ecotypes (LE1–LE9), capturing co‐associations between malignant and microenvironmental cell states, further refined prognostic classification, with LE1/LE2 and LE9 associated with the worst and most favourable prognosis, respectively. Both B‐cell states and LE retained independent prognostic value in multivariable analyses after adjusting for the cell of origin and the revised international prognostic index. Mutational profiling revealed state‐specific patterns: S1 harboured mutations in genes involved in epigenetic regulation, cytoskeletal organisation and tumour microenvironment (TME) interactions (*CREBBP*, *EZH2* and *ACTB*), whereas S5 was enriched for mutations promoting survival signalling, immune evasion and differentiation blockade (*MYD88*, *CD79B* and *TP53*). In vitro validation using human five DLBCL cell lines independently recapitulated the S1 and S5 transcriptomic states, supporting the existence of intrinsic, cell‐autonomous biological programs. Pathway analysis indicated that S1 was linked to cell adhesion, extracellular matrix remodelling and inflammatory signalling, while S5 displayed enrichment of cell‐cycle deregulation, metabolic reprogramming and immune evasion. Accordingly, S1 tumours appear reliant on TME interactions, whereas S5 tumours adopt a predominantly TME independent, intrinsically aggressive phenotype.

**Conclusions:**

Collectively, these findings delineate a biologically coherent framework linking pathway deregulation with cell state and ecotype‐specific mutational landscapes, refining DLBCL risk classification and adding an additional dimension to conventional prognostic models.

## Key points

- Applying the EcoTyper digital deconvolution framework to an independent DLBCL cohort supports the prognostic utility of B‐cell states and lymphoma ecotypes.
- Transcriptional and mutational profiles of S1 and S5 states suggest distinct microenvironment‐dependent and intrinsically aggressive tumor biology.
- This integrative analysis potentially complements standard clinical and genomic risk stratification models in DLBCL.

## Graphical abstract

Integrating DNA and RNA sequencing with digital deconvolution, this study identifies five malignant B‐cell states in DLBCL with distinct mutational and microenvironmental profiles. These states predict survival independently of standard indices, revealing a critical dichotomy between favourable TME‐dependent (S1) and aggressive TME‐independent (S5) phenotypes to refine precision medicine.

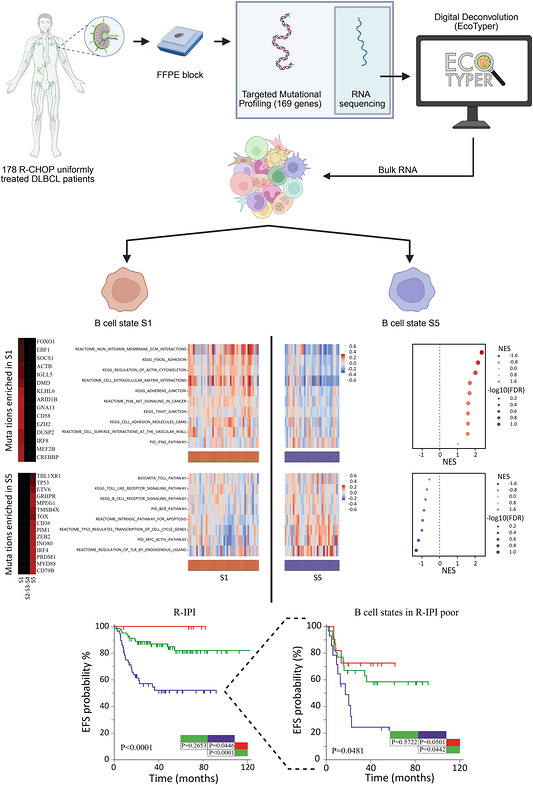

## 1 INTRODUCTION

Diffuse large B‐cell lymphoma (DLBCL) is a heterogeneous and clinically aggressive form of non‐Hodgkin lymphoma (NHL) accounting for approximately 30%‒40% of all NHL cases, and is characterised by the uncontrolled proliferation of large B lymphocytes within lymphoid tissues.^1^, ^2^, ^3^, ^4^, ^5^ The standard treatment of DLBCL remains chemo‐immunotherapy with R‐CHOP (rituximab, cyclophosphamide, doxorubicin, vincristine and prednisone).^6^, ^7^, ^8^ While this regimen is safe and effective, up to 30%–40% of patients eventually relapse.^6^, ^9^, ^10^ Therefore, improving the knowledge of DLBCL heterogeneity is crucial to identify high‐risk patients and develop more efficacious therapy regimens.^5^, ^11^, ^12^, ^13^, ^14^

Recent years have witnessed a rapid evolution in the understanding of the molecular landscape of DLBCL pathogenesis, revealing a complex interplay of genetic, molecular and microenvironmental factors highlighting unique genomic subtypes with important implications on targeted therapy.^3^, ^12^, ^15^, ^16^, ^17^, ^18^, ^19^, ^20^ DLBCL exhibits a crucial molecular and clinical divergence largely attributed to the cell of origin (COO) of the transformed B lymphocytes. Patients with the germinal centre B‐cell‐like (GCB) subtype have a better prognosis compared to those with an activated B‐cell‐like (ABC) subtype.^15^, ^16^, ^21^, ^22^, ^23^, ^24^, ^25^, ^26^ Alongside COO distinction, genomic analyses allowed the identification of several alterations, including point mutations, copy number variations and structural rearrangements, suggesting their implication in DLBCL clinical diversity. Particularly, mutational landscape analysis has revealed a panorama of driver mutations and oncogenic pathways that play pivotal roles in DLBCL risk stratification.^4^, ^27^, ^28^, ^29^, ^30^

Growing evidences further suggest that the tumour microenvironment (TME) is fundamental in shaping DLBCL complexity.^31^, ^32^, ^33^, ^34^, ^35^, ^36^, ^37^, ^38^, ^39^ In this context, Steen et al. have recently introduced the EcoTyper algorithm, a state‐of‐the‐art computational tool that operates as an integrated machine‐learning framework.^40^ This tool is primarily designed to provide high‐resolution identification of various cell types within the TME, including malignant B cells as well as non‐malignant immune and stromal cell populations.^40^ EcoTyper can identify unique molecular signatures associated with different cell states, and provide an ecosystem‐level perspective of the interactions between different cell types, unraveling the complex dynamics of DLBCL TMEs.

In this study, based on the framework established by Steen et al., our main objective is to provide a rigorous external validation and biological extension of these pre‐defined microenvironmental components. Specifically, we present: (i) a clinical validation of EcoTyper‐derived malignant B‐cell states in a real‐world cohort of newly diagnosed DLBCL patients uniformly treated with R‐CHOP; (ii) the correlation between transcriptionally defined cell states, lymphoma ecotypes (LEs), and established prognostic classifiers, including COO and revised international prognostic index (R‐IPI), highlighting their independent prognostic value; and (iii) an integrated RNA sequencing (RNA‐seq) and mutational profiling analysis that reveals state‐specific genetic landscapes and pathway‐level alterations, thereby expanding the biological and clinical understanding of DLBCL heterogeneity.

## 2 MATERIAL AND METHODS

### 2.1 DLBCL cohorts

The study included 204 formalin‐fixed paraffin‐embedded (FFPE) sections of lymph node (LN) samples from patients with positron emission tomography (PET) and computed tomography scan (CT) defined stage I‒IV DLBCL, not otherwise specified (NOS) as defined by the WHO.^41^ Inclusion criteria for all patients were: histopathological diagnosis of DLBCL NOS, no previous therapy, age >18 years, HIV negativity, treatment with R‐CHOP or R‐CHOP‐like regimens, staging and response evaluation by PET‒CT, availability of data regarding clinical laboratory features, treatment given and survival outcome. Exclusion criteria were based on: (i) high‐grade B‐cell lymphomas with MYC and BCL2 and/or BCL6 rearrangements (double‐hit/triple‐hit lymphomas),^41^ based on a thorough review of comprehensive medical records, physician referral letters, and available diagnostic cytogenetic data (Fluorescence In Situ Hybridization ‐ FISH) from the referring centres; (ii) histological transformation from indolent lymphoma or follicular lymphoma grade 3B; and (iii) post‐transplant lymphoproliferative disorder.

**FIGURE 1:**
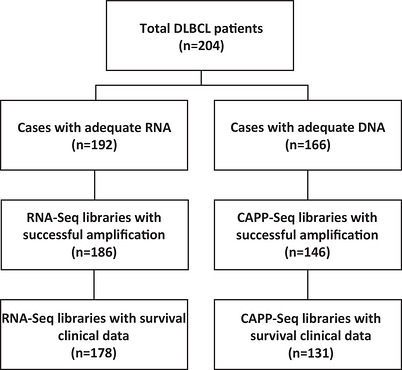
Flowchart of the study with the number of patients analysed.

All samples with inadequate RNA, RNA libraries, or no clinical data were excluded from the final cohort of 178 DLBCL patients (Figure 1). Comparison of baseline clinical characteristics between the excluded cases and the final cohort revealed no statistically significant differences, ruling out potential selection biases (data not shown). Clinical outcome data were updated as of September 2022. The median follow‐up from DLBCL diagnosis was 51.2 (95% confidence interval [CI] 44.9‒156.2 months). The R‐IPI, calculated by including age >60 years, stage III/IV disease, elevated lactate dehydrogenase (LDH) level, Eastern Cooperative Oncology Group (ECOG) performance status ≥2, more than one extranodal site (ENS) information at the time of diagnosis,^42^ was available for 143/178 patients. Table 1 summarises clinical and histopathological details of the whole cohort.

**TABLE 1:** Characteristics of the retrospective diffuse large B‐cell lymphoma (DLBCL) cohort.

Variable	Status	*N*	*n*	%
Age	≤60	178	97	54.5
	>60		81	45.5
Gender	Female	178	90	50.6
	Male		88	49.4
LDH	≥ULN	143	88	61.5
	<ULN		55	38.5
Ann Arbor stage	III‒IV	144	99	68.8
	I‒II		45	31.3
ECOG PS	≥2	144	16	11.1
	0‒1		128	88.9
ENS	≥2	144	36	25.0
	0‒1		108	75.0
B‐symptoms	Yes	145	45	31.0
	No		100	69.0
R‐IPI	Very good	143	7	4.9
	Good		80	55.9
	Poor		56	39.2
COO by Hans’ algorithm	GCB	167	71	42.5
	Non‐GCB		96	57.5
COO by Lymph2Cx	ABC	173	61	35.3
	GCB		81	46.8
	Unclassified		31	17.9
CD10	+	174	41	23.6
	–		133	76.4
BCL6	+	174	139	79.9
	–		35	20.1
MUM1	+	172	100	58.1
	–		72	41.9
BCL2	+	174	116	66.7
	–		58	33.3
MYC	+	173	25	14.5
	–		148	85.5
BCL2/MYC	BCL2–/MYC–	172	53	30.8
	BCL2–/MYC+		5	2.9
	BCL2+/MYC–		94	54.7
	BCL2+/MYC+		20	11.6
Response at the end of treatment	CR	178	138	77.5
	PR		25	14.0
	Non‐responder		15	8.4

*Note*: CD10, BCL6, MUM1, BCL2 and MYC staining was performed by immunohistochemistry.

Abbreviations: ABC, activated B‐cell; COO, cell of origin; CR, complete response; ECOG PS, Eastern Cooperative Oncology Group performance status; ENS, extranodal sites; GCB, germinal centre B‐cell; LDH, lactate dehydrogenase; PR, partial response; R‐IPI, revised international prognostic index; ULN, Upper Limit of Normal.

The use of clinical samples for this study was approved by the IRB of University of Modena (approval no. 44.2016) upon informed consent in accordance with the declaration of Helsinki.

### 2.2 Immunohistochemistry

Immunohistochemistry (IHC) was performed on 4 µm FFPE tissues. Slides, stained with haematoxylin‒eosin and/or Giemsa stain, and with the specific immunohistochemical markers, were reviewed by the pathologists (A.M., V.D. and A.D.N.). The minimum IHC panel included CD20, CD3, CD10, BCL6, MUM1/IRF4, BCL2, MYC and Ki67 (see Table S1 for the antibodies and clones list). When one or more immunostains were lacking, they were performed following local protocols on sections obtained from representative paraffin blocks.

### 2.3 Cell of origin classification

The classification of the COO was carried out using both IHC and the Lymph2Cx panel on the Nanostring platform. A positivity threshold greater than 30% of tumour cells staining was employed for CD10, BCL6 and MUM1/IRF4 markers, followed by Hans’ algorithm for categorisation of DLBCL cases in GCB and non‐GCB (Table 1).^24^ Based on the expression of 20 specific genes using the Lymph2Cx analysis on RNA extracted from FFPE samples, patients were subdivided into GCB, ABC and unclassified subsets (Table 1).^25^

The assessment of double‐expressor (DE) phenotype, based on the co‐expression of BCL2 and MYC, was established through IHC assessment of MYC (positive when ≥40%) and BCL2 staining (positive when ≥50%; Table 1).^43^

### 2.4 RNA sequencing

For patient samples, RNA was extracted from 10 mm unstained FFPE sections using either the Maxwell (Promega) or MagCore (RBC Bioscience) automated extractors. For cell lines, total RNA was isolated from five representative human DLBCL cell lines (SU‐DHL‐2, SU‐DHL‐8, SU‐DHL‐10, OCI‐Ly7 and RI‐1) using the AllPrep DNA/RNA Kit (Qiagen).

RNA integrity was evaluated using the Agilent Tapestation (Agilent Technologies). As expected due to the FFPE fixation protocol, the majority of tissue‐derived RNA showed an RNA integrity number (RIN) <3. Tissue RNA quality was further assessed using the transcript integrity number (TIN). For both patient and cell line samples, sequencing libraries were prepared using 150 ng of total RNA with the RNA HyperPrep with RiboErase (HMR) kit (Roche Sequencing Solutions), as detailed in the Supporting Information.

The sequencing datasets generated in this study have been deposited in the Gene Expression Omnibus (GEO) under the accession number GSE337531.

### 2.5 RNA‐seq analysis

RNA‐seq reads were pseudo‐aligned against the human transcriptome (GENCODE v.39) with Salmon obtaining a median of over 80% of mapped rate (Figure S2A).^44^ Differential expression analysis was performed using the edgeR package with standard parameters.^45^ Further analyses are described in the Supporting Information.

For patient samples, transcriptomic data were used to quantify TME abundance and define cell state and LE via EcoTyper (https://EcoTyper.stanford.edu/).^40^

Concurrently, to evaluate the enrichment of B‐cell state profiles and downstream oncogenic pathways across the transcriptomic profile of DLBCL cell lines, single‐sample gene set enrichment analysis (ssGSEA) was implemented using the Python gseapy library. Briefly, cell line analysis employed the top 1% marker gene signatures defining each EcoTyper B‐cell state, alongside a curated compendium of canonical DLBCL biological networks. The resulting normalised enrichment scores underwent row‐wise *Z*‐score transformation to capture and visualise relative transcriptomic enrichment variations across the models.

### 2.6 Targeted sequencing

Cancer personalised profiling by deep sequencing (CAPP‐seq) panel seed genes were chosen based on the frequency of mutation in B‐cell tumours, resulting in a total of 169 genes (Table S2). Library preparation was performed starting from 100 ng of DNA extracted from FFPE, and following the Kapa HyperPrep Kit (ROCHE) instructions, as described in the Supporting Information. Targeted locus sequencing was performed with a minimum depth of coverage of 100× per locus. Somatic variant calling was performed utilising the LoFreq pipeline. Only mutation with a variant allele frequency (VAF) >5% and with a Rare Exome Variant Ensemble Learner (REVEL)^46^ score greater than .4 were retained. Detected variants were rigorously filtered against the dbSNP database. Genetic subgrouping was performed using the LymphGen^29^ algorithm tool, utilising somatic mutations. For further details, see Supporting Information.

### 2.7 Statistical analysis

All statistical analyses were performed using MedCalc Statistical Software version 22.014 (MedCalc Software Ltd) and the R statistical computing environment (R Foundation for Statistical Computing). The associations between clinical presentation and molecular/pathological characteristics were assessed by chi‐square or Fisher's exact test for nominal data and Wilcoxon rank‐sum test (Mann‒Whitney *U* test) for continuous variables. Comparison across group were performed via a non‐parametric Kruskal‒Wallis test. Event‐free survival (EFS) was measured from the date of initial diagnosis to the date of therapy failure, disease progression, relapse or death (Figure S1A). Overall survival (OS) was measured from the date of initial diagnosis to the date death or last follow‐up. Median follow‐up was calculated using the reverse Kaplan‒Meier method. Multivariate analyses to identify variables with independent prognostic significance were carried out by a Cox regression model; cases with missing clinical covariates were handled by complete case analysis. To prevent overfitting, and stabilise hazard ratios in multivariable analyses involving small sub‐partitions a penalised Cox proportional hazards regression model employing a Ridge (L2) penalty was used.^47^ Internal validation and coefficient stabilisation were subsequently performed via bootstrap resampling with 1000 iterations, from which empirical 95% confidence intervals were derived. Molecular studies were blinded to the study end points.

## 3 RESULTS

### 3.1 DLBCL cohort

Among 178 patients (81 aged >60 years at the time of diagnosis; 90 female), 52 progressed and among them 33 died after the end of therapy (Table 1). Based on R‐IPI stratification, patients were categorised into three distinct risk subgroups: very good (seven patients), good (80 patients) and poor (56 patients; Table 1). In keeping with previous reports,^42^ patients in the poor risk group (mean 47.3 months) presented significant shorter EFS than patients in the very good risk group (mean 78.3 months; *p* < .0001) and good risk group (mean 98.7 months; *p* = .0459; Figure S1B). Despite a trend toward a better outcome, no significant difference was observed between the very good and good risk groups, likely due to the small sample size of the very good risk group (Figure S1B).

### 3.2 RNA sequencing

Since our libraries were generated from RNA extracted from FFPE material, we first assessed the quality of our gene expression data using the previously established Lymph2Cx classification as a reference. As reported in Table 1 and Supporting Information, the Lymph2Cx assay identified 81 cases as GCB, 61 as ABC and 31 (17.9%) as unclassified. Quality assessment of RNA‐seq data was performed by analysing the expression levels of the 15 genes included in the Lymph2Cx signature (*PIM2*, *ASB13*, *S1PR2*, *CCDC50*, *CYB5R2*, *SERPINA9*, *ITPKB*, *CREB3L2*, *MME*, *IRF4*, *TNFRSF13B*, *RAB29*, *MAML3*, *LIMD1* and *MYBL1*)^25^ solely in the 142 cases classified as ABC or GCB by Lymph2Cx. Consistent with the Lymph2Cx classification, hierarchical clustering based on RNA‐seq expression values accurately separated ABC and GCB subtypes, showing high concordance with the Nanostring procedure (.97 kappa Cohen), thereby confirming the ability of the RNA‐seq to recapitulate the molecular information obtained with Lymph2Cx (Figure S2B).

To further assess RNA‐seq data quality, we compared the gene expression profile of 61 ABC cases versus 81 GCB cases, as defined by both Lymph2Cx and RNA‐seq, identifying 1089 differentially expressed genes. Among these, 209 genes were upregulated in ABC and 880 in GCB samples.

Confirming the fidelity of our FFPE‐derived RNA‐seq data gene set variation analysis (GSVA) and GSEA revealed multiple gene sets previously shown to be enriched in either GCB or ABC subtypes (Figure S2C).^15^, ^21^

### 3.3 EcoTyper

Having validated our RNA‐seq data, we used the machine‐learning approach of EcoTyper for dissecting the cellular heterogeneity of our DLBCL cohort.^40^ Digital deconvolution was calculated by EcoTyper algorithm on 178 samples with clinical data (Table 1). EcoTyper demonstrated efficacy in distinguishing a total of 13 distinct cell types, including B cells and additional 12 cell types associated with the TME. For each cell type, EcoTyper characterised the specific transcriptional programs termed ‘cell states’ (Table S3).

### 3.4 B‐cell state

**FIGURE 2:**
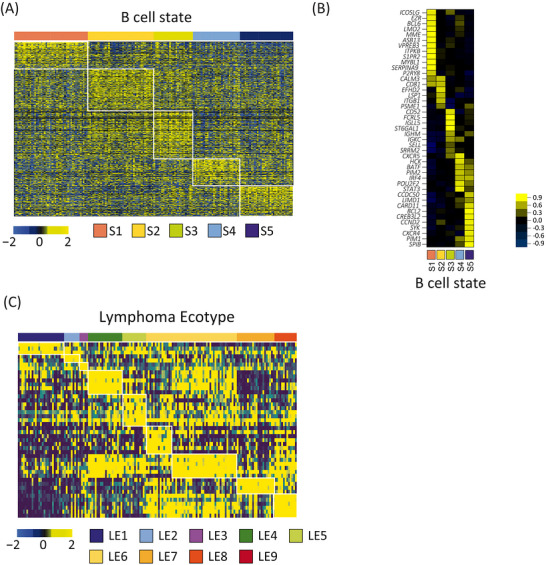
EcoTyper classification of diffuse large B‐cell lymphoma (DLBCL) cohort. (A) Heatmap depicting the five B‐cell states (S1‒S5) identified by transcriptomic analysis of 178 DLBCL patients. Patient samples (columns) are organised by the most prevalent cell state. (B) Heatmap showing the normalised expression of genes defining the specific transcriptional signature mirroring the maturation processes of B cells from germinal centre to plasma cell as exploited by EcoTyper. (C) Heatmap depicting the nine lymphoma ecotypes states (LE1‒LE9) identified by transcriptomic analysis of 178 DLBCL patients. Patient samples (columns) are organised by the most prevalent cell state.

Based on specific differentiation program of B cell, five different cell states named from S1 to S5 were detected (Figure 2A and Table S3). Importantly, the TIN scores were uniformly distributed across the five B‐cell states (*p* = .076), confirming that the transcriptomic signatures is unrelated either to technical biases or RNA fragmentation. In keeping with previous findings, S1 exhibited a transcriptional signature characteristic of germinal centre cells, featuring elevated expressions of genes such as *MME*, *SERPIN9* and *BCL6*, while S5 displayed a plasma cell signature with an overexpression of *IRF4*, *CXCR4* and *PIM1* (Figure 2B).^40^ Likewise, the remaining cell states (S2, S3 and S4) showed unique gene programs mirroring the maturation processes of B cells from germinal centre to plasma cell as exploited by EcoTyper (Figure 2A,B). A comparison between our cohort and the training cohort provided by EcoTyper (438 DLBCL samples) revealed a remarkably similar expression profile (Figure S3A). Patients were assigned to the specific B‐cell state (S1–S5) showing the highest relative abundance score according to the EcoTyper algorithm resulting in the identification of 46, 42, 23, 29 and 36 DLBCL cases to the S1, S2, S3, S4 and S5 B‐cell state, respectively, with two cases not assigned (Table S3).

**FIGURE 3:**
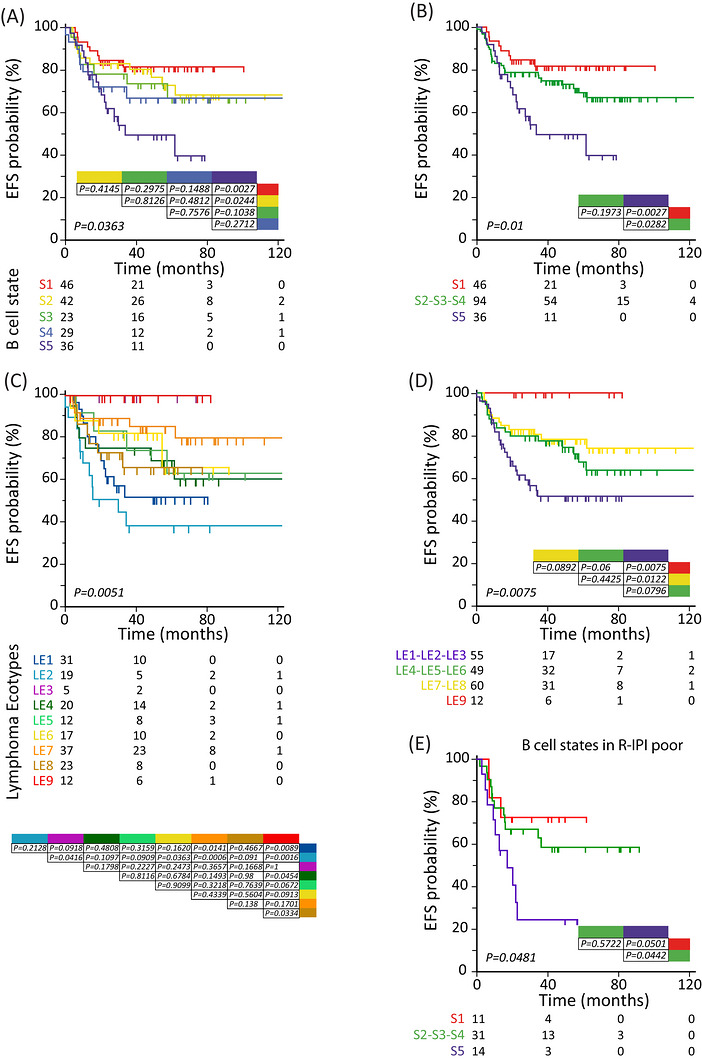
B‐cell states and lymphoma ecotype (LE)‐associated survival curves. (A) Kaplan‒Meier curves comparing event‐free survival (EFS) probabilities of 176 diffuse large B‐cell lymphoma (DLBCL) patients according to B‐cell state classification. The graph shows 46 S1 (red line), 42 S2 (yellow line), 23 S3 (green line), 29 S4 (blue line) and 36 S5 (purple line) DLBCL cases. (B) Kaplan‒Meier curves comparing EFS probabilities 176 DLBCL patients grouped according to recursive partitioning of B‐cell state classification. The graph shows 46 S1 (red line), 94 S2‒S3‒S4 (green line) and 36 S5 (purple line) DLBCL cases. (C) Kaplan‒Meier curves comparing EFS probabilities of 176 DLBCL patients according to LE classification. The graph shows 31 LE1 (blue line), 19 LE2 (light blue line), five LE3 (light purple line), 20 LE4 (dark green line), 12 LE5 (light green line), 17 LE6 (yellow line), 37 LE7 (orange line), 23 LE8 (ochre line) and 12 LE9 (red line) DLBCL cases. (D) Kaplan‒Meier curves comparing EFS probabilities 176 DLBCL patients grouped according to recursive partitioning of LE cell‐state classification. The graph shows 55 LE1‒LE2‒LE3 (purple line), 49 LE4‒LE5‒LE6 (green line), 60 LE7‒LE8 (yellow line) and 12 LE9 (red line) DLBCL cases. (E) Kaplan‒Meier curves comparing EFS probabilities of 56 DLBCL patients divided by partitioned B‐cell state in the context of revised international prognostic index (R‐IPI) ‘poor’ cases only. The graph shows 11 S1 (red line), 31 S2‒S3‒S4 (green line) and 14 S5 (purple line) DLBCL cases. The number of patients in each group is reported. *p*‐Values refer to log‐rank test.

As reported in Figure 3A, S1 patients presented the longest EFS, and S5 the shortest, while similar behaviour was observed for S2, S3 and S4 patients who presented an intermediate outcome (Figure 3A). Bootstrap analysis confirmed a significantly worse clinical outcome for S5 patients (median hazard ratio [HR] 3.00; 95% CI 1.41‒6.30; *p* = .002) alongside a progressive risk increase from S1 to S5, validating a biologically driven stratification. Based on this observation, we applied an exploratory, data‐driven strategy to partition the B‐cell state classification into three broader groups: S1 (*n* = 46), S2‒S3‒S4 (*n* = 94) and S5 (*n* = 36). Consistently, S5 B‐cell state patients presented significantly shorter 3‐year EFS (50%) than patients classified as S2‒S3‒S4 (74%; *p* = .0195) and as S1 (82%; *p* = .0029; Figure 3B).

Correlating the co‐occurrence of the B‐cell states with the COO as established by Lymph2Cx (Figure S4A), the GCB subgroup was enriched in S1 (55%; 45/81) and S2 (26%; 21/81) cases, whereas S3, S4 and S5 were scarcely represented (11%, 3% and 3%, respectively), in keeping with the fact that S1‒S2 cases usually reflect the transcriptional program of germinal centre cells (Table S3). On the contrary, ABC subtype was enriched in S4 (29%; 18/61) and S5 (46%; 28/61) cases, being these cell states mostly related to post‐germinal centre cells signature (Table S3 and Figure S4A).

As shown in Figure S5, no significant association was observed between EFS and the various cell states other than B cell. Among them, the dendritic cell state S1 appeared to be associated with longer EFS compared to S3 and S4 cell states.

### 3.5 TME‐associated cell states (LE)

In addition to the B‐cell states, EcoTyper allowed the recognition of other different cell states detecting different cell programs in the context of all the cell types of the TME: dendritic cells, endothelial cells, fibroblasts, mast cells, neutrophils, natural killer (NK) cells, plasma cells, CD4 T cells, monocytes and macrophages, CD8 T cells, CD4 follicular helper T cells and CD4 regulatory T cells. Figure S5 shows the clinical impact on EFS of the different TME cell states highlighting no evident clinical correlation.

According to the different cell states derived from specific cell types, distinct association patterns of cell states were identified through the Jaccard index (Figure S6) in keeping with original data.^40^ As an example, neutrophil state S1, CD8+ T‐cell state S4, CD4+ T‐cell state S2 and monocyte state S3 were consistently observed to coexist in the microenvironment, reflecting non‐random associations. Among B‐cell states, S1 exhibited the highest levels of interaction with plasma cell state S3, fibroblast state S2, regulatory CD4+ T‐cell state S2, highlighting a microenvironment‐dependent behaviour of cell states (Figure S6).

EcoTyper, by maximising these co‐association patterns into distinct communities termed Lymphoma Ecotypes (LEs), which represents the TME‐informed lymphoma subgroups, identified nine LEs associated with clear variations in molecular characteristics (Figure 2C). In keeping with B‐cell states, no significant difference in TIN scores were observed across the LE classifications (*p* = .1393). In addition, LEs classification demonstrated an EFS gradient from LE1 to LE9, with the LE1 and LE2 showing the worst outcome (Figure 3C). Bootstrap approach confirmed that LE2 and LE1 were significantly associated with an inferior EFS (median HR 3.52, 95% CI 1.77‒6.30, *p* < .001; median HR 2.25, 95% CI 1.26‒3.70, *p* = .014, respectively). Interestingly, LE7 displayed good prognosis although in our cohort was constituted by 26% of ABC cases according to Lymph2Cx classification (Supporting Information, Figure S4B and Table S3). As observed in Figure 3D, an exploratory recursive partitioning of LEs delineated four patient subsets characterised by significant differences in terms of survival outcomes.

### 3.6 OS analysis

Consistent with the EFS findings, OS analyses revealed similar prognostic trajectories across the identified subsets. As shown in Figure S9, OS did not reach statistical significance in the high‐resolution partitions for either the B‐cell states (*p* = .1872 or the LE (*p* = .162). Notably, only the grouped three‐tier B‐cell state model maintained significance for OS (*p* = .0489; Figure S9).

### 3.7 Multivariate models

As detailed above, the B‐cell states and LE subgroups effectively classify DLBCL patients into three or four distinct categories. When evaluated in two different multivariate models (*n* = 140) along with R‐IPI and COO, they remained independent EFS predictors (B‐cell state, *p* = .009; LE, *p* = .022) of poor outcome along with R‐IPI (*p* = .0002 and .001; Table 2). Importantly, within the penalised multivariable bootstrap analysis, the S5 state maintained a strong independent association with inferior EFS (*p* = .051; Table 2). In a parallel analysis, the grouped LE1‒LE2‒LE3 ecotypes emerged again as statistically significant independent predictor of inferior EFS when compared to the LE9 reference (*p* = .016; Table 2). Notably, B‐cell states offered further stratification within the R‐IPI poor patient group (*N* = 56). As shown in Figure 3E, the S1 B‐cell state identified a subgroup with an EFS outcome similar to that of R‐IPI very good/good cases, whereas the S5 B‐cell state was associated with the poorest outcomes. After correcting for the limited subgroup sample size, the S5 state maintained a statistically significant association with inferior EFS, yielding a median HR of 2.70 (95% CI 1.10‒7.08, *p* = .034) when compared to the S1 reference.

**TABLE 2:** Univariable and multivariable analyses of event‐free survival (EFS) (*n* = 140).

	UVA				MVA with B‐cell state				Bootstrapped penalised Cox regression (B‐cell state)				MVA with lymphoma ecotypes				Bootstrapped penalised Cox regression (lymphoma ecotypes)			
	HR	LCI	UCI	*p*‐Value	HR	LCI	UCI	*p*‐Value	Median HR	LCI	UCI	*p*‐Value	HR	LCI	UCI	*p*‐Value	Median HR	LCI	UCI	*p*‐Value
R‐IPI (poor)	4.13	2.07	8.25	***<.001***	3.69	1.84	7.39	***.002***	2.77	1.61	5	***<.001***	3.21	1.56	6.59	***.001***	2.58	1.45	4.64	***<.001***
COO nanostring (ABC)	2.74	1.47	5.10	***.002***	ni				1.51	.82	2.87	.192	ni				1.57	.81	3.13	.184
COO nanostring (unclassified)	1.36	.58	3.17	.477	–				1.5	1.69	2.95	.312	–				1.49	.63	3.27	.336
B‐cell state (S2‒S3‒S4)	1.66	.75	3.70	.2	–				1.19	.66	2.14	.614	Not used							
B‐cell state (S5)	3.27	1.40	7.59	***.006***	2.41	1.21	4.78	***.012***	2.02	.98	3.89	***.051***	Not used							
Lymphoma ecotype (LE1‒ LE2‒ LE3)	2.15	1.52	8.90	***.01***	Not used								2.18	1.11	4.26	***.022***	1.87	1.11	3.27	***.016***
Lymphoma ecotype (LE4‒LE5‒LE6)	1.76	.75	6.52	*.15*	Not used								ni				1.62	.87	2.75	.128
Lymphoma ecotype (LE7‒LE8)	1.52	.62	4.92	*.345*	Not used								ni				.94	.51	1.49	.798

*Note*: For MVA, median HRs, 95% CIs and empirical *p*‐values were derived from a 1000‐iteration bootstrap resampling procedure applied to a Ridge‐penalised Cox proportional hazards model to ensure robust estimations. B‐cell state was identified by EcoTyper. Symbol ‘‒’ denotes variable with *p* > .1 in UVA removed from the MVA model. Statistically significant *p*‐values (*p* < .05) are indicated in bold and italics.

Abbreviations: CI, confidence interval; COO, cell of origin according to Lymph2Cx; HR, hazard ratio; LCI, 95% lower CI; MVA, multivariable analysis; ni, excluded by the final model after stepwise MVA; R‐IPI, revised international prognostic index; UCI, 95% upper CI; UVA, univariable analysis.

### 3.8 Genetic mutations and profiles

**FIGURE 4:**
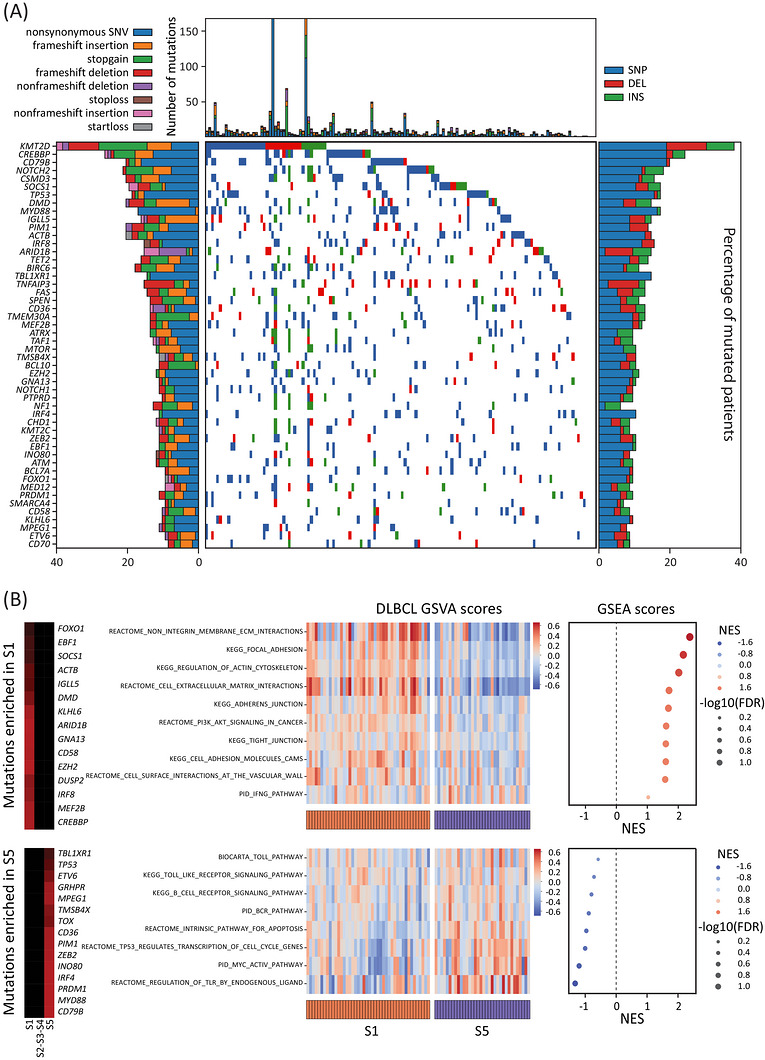
Molecular characterisation of diffuse large B‐cell lymphoma (DLBCL). (A) Case‐level genomic profile of the 146 DLBCL patients analysed through cancer personalised profiling by deep sequencing (CAPP‐seq). Each column represents one patient and each row represents genomic abnormalities. (B) Differential pathway usage between S1 and S5 B‐cell state cases, calculated considering only pathway including at least two mutated genes for each B‐cell state. Left panel reports the heatmap of significant gene set variation analysis (GSVA) enrichment scores for S1/S5 in DLBCL bulk RNA sequencing (RNA‐seq) data. The right Dotplot reports gene set enrichment analysis (GSEA) normalised enrichment scores scaled by ‒log10(FDR).

Mutational analysis on 146 samples identified a total of 1.819 mutations, with a mean mutation rate of 8 (range: 0–83; Figure 4A). In keeping with literature,^4^, ^28^, ^29^, ^48^ among the most frequently mutated genes we observed *KMT2D*, *CREBBP*, *CD79B*, *NOTCH2*, *CSMD3*, *SOCS1*, *TP53*, *MYD88*, *IRF8* and *ACTB* (Figure 4A). Notably, no significant differences in terms of EFS were observed between cases harboring mutations versus wild‐type cases within the top 15 most frequently mutated genes, even when stratified by COO classification (Table S4).

By feeding the LymphGen tool,^29^ 79 (54%) patients were assigned to a molecular subtype while the remaining 67 (46%) patients were classified as ‘other’ (Figure S7A), in keeping with reported data.^29^ Specifically, 18 (12%) patients were classified as MCD, 20 (14%) as ST2, 16 (11%) as EZB, three (2%) as N1, and 22 (15%) as BN2 (Figure S7A). Excluding patients in the ‘other’ subgroup and those with no clinical data, MCD subgroup presented the poorest clinical outcome along with BN2 subgroup (Figure S7B).

In the absence of a clear association between LymphGen groups and B‐cell states, as highlighted in Figure S7C, according to an in‐house pipeline based on *Z*‐score, we identified specific pattern of mutation for each B‐cell states (Figure S8A). Based on the three groups partitioning of B‐cell states (Figure 3B) and on genes mutated in over 5% of cases, we identified distinct mutational landscapes clearly associated with the EcoTyper classification (Figure S8B). Specifically, the S1 B‐cell state (30 cases) was enriched for mutations in genes involved in transcriptional regulation (*CREBBP* and *EZH2*), B‐cell differentiation (*MEF2B*, *FOXO1* and *EBF1*) TME interaction modulation (*CD58* and *IRF8*), cytoskeletal dynamics and structural integrity regulation (*ACTB* and *DMD*). Conversely, the S5 B‐cell state (28 cases) exhibited a mutational profile characterised by alterations in genes linked to survival signalling (*TP53* and *PIM1*), block of the B‐cell differentiation (*TBL1XR1*, *IRF4* and *PRDM1*) immune signalling pathways (*CD79B* and *MYD88*).

Given their prognostic relevance, we focused only on B‐cell state S1 and S5 to evaluate the influence of mutations on gene expression dysregulation. Accordingly, we performed RNA‐seq GSVA and GSEA using the sets of mutated genes identified within each cell state (Figure 4B). Pathways associated with the specific mutations of B‐cell state S1 presented enrichment in processes related to cellular adhesion, mobility and interactions with the TME, highlighting disruptions in extracellular matrix dynamics and cytoskeletal organisation (Figure 4B). Enhanced responsiveness to environmental stimuli, along with overrepresented epigenetic regulation and chromatin remodeling pathways, further characterised this state (Figure 4B). On the other hand, in genesets linked to the specific mutations of B‐cell state S5, those driving aggressive proliferation were predominant, marked by deregulated cell‐cycle control and checkpoint evasion. Chronic activation of proliferative signalling, immune evasion mechanisms, metabolic alterations and resistance to apoptosis were also prominent features (Figure 4B).

### 3.9 Orthogonal validation of B‐cell states features in DLBCL cell lines

We applied a computational deconvolution pipeline to bulk RNA‐seq data from a panel of five human‐derived DLBCL cell lines. As shown in Figure S10A, we identified at least one cell line resembling each specific B‐cell state except for S2 (with SU‐DHL‐10, SU‐DHL‐8, SU‐DHL‐2 and RI‐1 corresponding to S1, S3, S4 and S5, respectively), whereas OCI‐Ly7 did not clearly correlate with any single state.

To validate the transcriptional differences identified between patient derived S1 and S5 profiles, we performed ssGSEA. SU‐DHL‐10 presented a strong enrichment for GC B‐cell programs, retaining intrinsic transcriptional signatures associated with immune crosstalk, such as resting NK cell and specific T‐cell interaction profiles (Figure S10B). Conversely, RI‐1 exhibited a shift toward an ABC‐DLBCL phenotype, characterised by an enrichment of chronic BCR signalling, canonical NF‐κB activation and terminal differentiation pathways driven by IRF4 and MYD88/interferon networks (Figure S10B).

## 4 DISCUSSION

Expanded knowledge of the molecular pathogenesis, immunophenotypic spectrum and clinical outcome of DLBCL has been translated into several risk stratification models.^15^, ^23^, ^24^, ^28^, ^29^, ^40^ Additionally, categorisation of DLBCL into specific genetic subgroups through molecular profiling has enabled the design of target treatment approaches.^49^ Nevertheless, several essential aspects for a comprehensive understanding of DLBCL molecular heterogeneity remain unresolved.^8^, ^13^, ^33^ This study leverages advanced genomic and transcriptomic methodologies, incorporating modern deconvolution algorithms, to dissect the complexity of DLBCL within a well‐characterised clinical cohort.

Our data provide an independent validation of the study by Steen et al., confirming the existence of five malignant B‐cell states (S1–S5) with distinct transcriptional signatures and prognostic implications. In line with the original findings, S1 was associated with favourable outcome, while S5 identified patients with dismal prognosis. Although the grouping of intermediate states requires external confirmation to exclude data‐dependent fitting, our cell line RNA‐seq data revealed overlapping *Z*‐score distributions across the S2, S3 and S4 states, indicating that these intermediate classifications share highly similar transcriptional profiles and probably represent a biological continuum that support their consolidation alongside the distinct S1 and S5 prognostic groups.

Notably, this study reveals a previously unrecognised finding: B‐cell states classification enables further stratification within the R‐IPI poor‐risk group, with patients classified as S1 exhibiting EFS outcome comparable to those of R‐IPI very good/good cases. Furthermore, our analysis demonstrated for the first time that S1 and S5 represent independent prognostic factors of EFS beyond established classifiers such as R‐IPI and COO, underlying the critical role of molecular pathogenesis in shaping treatment response. As expected, salvage therapies at relapse and competing mortality risks inherent to this elderly population likely attenuated the prognostic value of OS in our model. This external validation in a real‐world R‐CHOP‐treated cohort highlights the robustness of EcoTyper as a clinically meaningful classifier and supports its potential clinical translation.

Collectively, these findings raise fundamental questions about the cellular and molecular mechanisms underlying these distinct phenotypes. To address these questions and provide a genomic extension to the original transcriptomic framework, we integrated the pre‐defined B‐cell states with mutational profiling using a panel of 169 recurrently mutated genes in DLBCL. Interestingly, the S5 subtype was characterised by transcriptional profiles associated with disrupted cell‐cycle regulation, features suggestive of immune evasion and increased invasiveness. Its mutational profile revealed alterations in *MYD88*, *CD79B* and *TP53* genes, which support chronic activation of proliferative and anti‐apoptotic pathways that could drive tumour growth and therapy resistance.^50^, ^51^, ^52^, ^53^ Synergistically, *TP53* mutations contribute to checkpoint evasion and genomic instability, while *MYD88* and *CD79B* mutations sustain NF‐κB‐mediated pro‐survival signalling, further reinforced by *PIM1*‐driven MYC transcriptional programs.^50^, ^51^, ^52^, ^53^ Mutations in *TBL1XR1* mutations likely disrupt the SMRT/NCOR complex, impairing plasma cell differentiation and promoting extranodal dissemination.^54^

In contrast, the S1 B‐cell exhibited enrichment of mutations in genes related to motility, transcription remodelling and inflammation. Specifically, *ACTB* mutations may alter the cytoskeleton, facilitating adhesion and controlling motility‐key processes for germinal centre dynamics.^55^, ^56^ *CREBBP* and *EZH2* mutations suggest significant epigenetic reprogramming, potentially enhancing transcriptional plasticity allowing cells to adapt to the microenvironment, while preserving proliferative behaviour.^57^, ^58^, ^59^, ^60^ Additionally, mutations in *IRF8*, *CD58* and *SOCS1* may modulate cytokine signalling to maintain inflammatory balance within the TME, while *IRF8* and *CD58* alterations suggest a deregulation of Th1 polarisation and impaired antigen presentation, potentially affecting antitumour immune responses.^61^, ^62^, ^63^, ^64^, ^65^ Although these mutations and their associated gene expression programs reflect relevant molecular alterations in S1, they are potentially insufficient to support full microenvironmental independence. In contrast to the S5 state, the S1 profile is not prominently enriched for immune‐evasive signatures, tracking with a lower apparent propensity to subvert immune surveillance mechanisms.^40^ Accordingly, despite the presence of oncogenic mutations, the relative absence of highly aggressive features together with the preservation of immune recognition may contribute to the more favourable clinical outcomes observed in S1 patients receiving anti‐CD20 monoclonal antibody therapy.

The functional mutation dichotomy observed between the S1 and S5 states was further supported by GSVAs involving the mutated genes. The S1 cell state was enriched for pathways related to cellular adhesion, extracellular matrix dynamics, and epigenetic regulation. These findings are supported by the co‐occurrence patterns showing that S1 B‐cell states are associated with pro‐inflammatory TME components, suggesting that dynamic microenvironmental interactions are closely linked to tumour behaviour in these patients. In contrast, S5 exhibited upregulation of proliferative signalling, cell‐cycle dysregulation and immune evasion pathways, consistent with a more cell‐autonomous and aggressive transcriptomic phenotype characterised by lower inferred T‐cell infiltration. This observation aligns with recent spatial transcriptomic findings demonstrating that T‐cell exclusion in aggressive B‐cell lymphomas can be actively sustained by ATR‐dependent DNA damage response and chromatin compaction programs, providing a potential biological framework aligning cell‐cycle deregulation with the transcriptomic features observed in S5.^66^ Furthermore, this aggressive phenotype is supported by the enrichment of *MYD88* and *CD79B* mutations, which are known to drive constitutive BCR signalling and NF‐κB activation, promoting lymphoma cell survival.^28^, ^29^, ^40^ All together, these data suggest that TME composition may further refine risk stratification beyond tumour‐intrinsic features alone.

In vitro validation across five DLBCL cell lines recapitulated the S1 and S5 dichotomy even in the absence of an active TME. Specifically, the S1 cell line maintained GC B‐cell features and intrinsic signatures associated with NK and T‐cell crosstalk, indicating a baseline molecular programming predisposed towards TME dependence. This aligns with the co‐occurrence of pro‐inflammatory TME components and high immune surveillance observed clinically in S1 patients. Conversely, the ABC‐like S5 cell line exhibited a microenvironment‐independent phenotype driven by chronic BCR signalling, canonical NF‐κB activation and MYD88/interferon networks. This reliance on cell‐autonomous survival cascades mirrors the mutational enrichment (e.g., MYD88, CD79B) and cell‐cycle dysregulation of our primary cohort, providing a biological rationale for the aggressive, immune‐evasive nature and poor prognosis of the S5 state. While these static models lack full cellular heterogeneity, future spatial transcriptomics, 3D co‐cultures or in vivo models will help fully validate these microenvironmental dynamics.

Recent advances in immunotherapy have highlighted the potential role of CD3×CD20 bispecific antibodies, which have demonstrated high response rate in relapsed/refractory DLBCL.^67^, ^68^, ^69^, ^70^ The S5 state showed reduced endogenous T‐cell infiltration, as indicated by its poor correlation index with T‐cell states. In this context, we hypothesise that CD3×CD20 bispecific antibodies, which recruit cytotoxic T cells and restore immune recognition, could be investigated as a therapeutic strategy for this subgroup, although this requires validation in clinical trials. Furthermore, ongoing clinical trials are evaluating R‐CHOP‐X approaches, where ‘X’ may represent an additional targeted agent selected based on molecular features.^49^ Following on this rationale, EZH2 or histone deacetylase inhibitors, which reshape transcriptional programs and enhance sensitivity to chemoimmunotherapy, could be considered for S1 patients with an epigenetically plastic phenotype. In contrast, S5 patients, characterised by chronic NF‐κB and BCR signalling activation, might be candidates for investigation with BTK or MALT1 inhibitors specifically targeting these aberrant survival signalling pathways.

Of note, EcoTyper also infers specific LEs. In this study, the LE4 ecotype, enriched in immune‐reactive TME components, might be particularly responsive to agents that potentiate pre‐existing cytotoxic T‐cell activity, such as immune checkpoint inhibitors or CD20×CD3 bispecific antibodies, thereby amplifying antitumour immune responses. Conversely, LE1/LE2 ecotypes, which exhibit immunosuppressive features and an aggressive phenotype, may require combination strategies targeting both tumour‐intrinsic drivers and the immunosuppressive microenvironment.^39^, ^40^, ^69^

Approximately 60% of cases could be categorised into a specific R‐CHOP‐X treatment group based on mutational profiling.^49^ However, RNA‐seq enables a more comprehensive classification by capturing transcriptional patterns that align with mutational defined categories, even in cases lacking specific genetic alterations. This suggests that transcriptomic approaches can identify pathway dysregulation not evident at the mutational level but functionally equivalent to alterations observed in genetically defined subgroups. This capability underscores the value of transcriptomic state‐level analysis in refining patient stratification and complementing existing models, ultimately enhancing precision medicine approaches in DLBCL treatment. A possible limitation of our study is the constrained statistical power in certain subgroup analyses. Specifically, partitions yielding small subsets, such as the LE9 ecotype (*n* = 12) or the S1 state within the R‐IPI poor cohort (*n* = 11), inherently carry a risk of model overfitting and unstable hazard estimates. Nevertheless, these results highlight promising biological trends that mandate validation in larger, independent real‐world cohorts.

## AUTHOR CONTRIBUTIONS

Robel Papotti and Filippo Vit performed molecular studies, interpreted data and wrote the manuscript. Tamara Bittolo and Elisa Forti performed and interpreted molecular studies, and contributed to data interpretation. Filippo Vit and Riccardo Bomben generated the bioinformatics pipeline of analysis, and performed statistical analyses. Federico Pozzo, Stefania Bettelli, Samantha Pozzi, Luca Braglia, Arianna Di Napoli, M. Christina Cox, Tamar Tadmor, Giovanna R. Mansueto, Pellegrino Musto, Leonardo Flenghi, Martina Quintini, Sara Galimberti, Valentina Donati, Michele Spina, Alberto Zamò, Andreas Rosenwald and Stefano Sacchi collected clinical data and contributed to data interpretation. Valter Gattei, Stefano Sacchi and Riccardo Bomben designed the study, interpreted data and wrote the manuscript.

## CONFLICT OF INTEREST STATEMENT

The authors declare they have no competing financial interests.

## ETHICS STATEMENT

The use of clinical samples for this study was approved by the IRB of University of Modena (Approval n. 44.2016) upon informed consent in accordance with the declaration of Helsinki.

## Supporting information



Supporting Information

Supporting Information

Supporting Information

Supporting Information

Supporting Information

Supporting Information

Supporting Information

Supporting Information

Supporting Information

Supporting Information

Supporting Information

Supporting Information

Supporting Information

Supporting Information

Supporting Information

## Data Availability

The datasets generated and/or analysed during the current study will be available from the corresponding authors upon request on the Gene Expression Omnibus (GEO) under the accession number GSE337531.
