# Supplementary material for: Dissecting diffuse large B‐cell lymphoma heterogeneity: New insights from a state‐specific molecular characterisation
Source: Clin Transl Med. 2026 Aug 19;16(8):e70757. doi: 10.1002/ctm2.70757 (PMC13487464; doi:10.1002/ctm2.70757)
Supplement: Supplementary file 1 [file CTM2-16-e70757-s001.pdf]

Figure S4

A

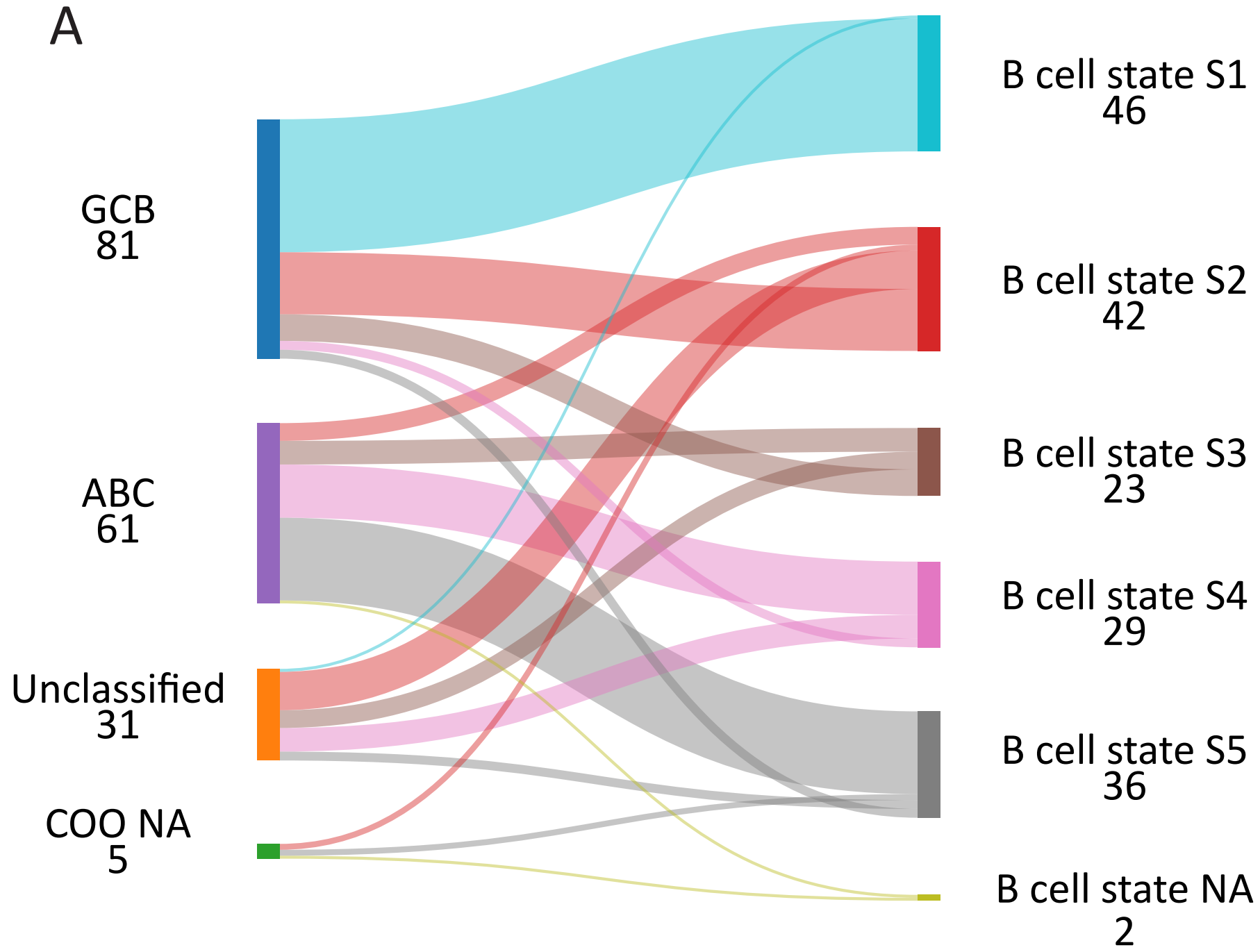

GCB
81
ABC
61
Unclassified
31
COO NA
5
B cell state S1
46
B cell state S2
42
B cell state S3
23
B cell state S4
29
B cell state S5
36
B cell state NA
2

B

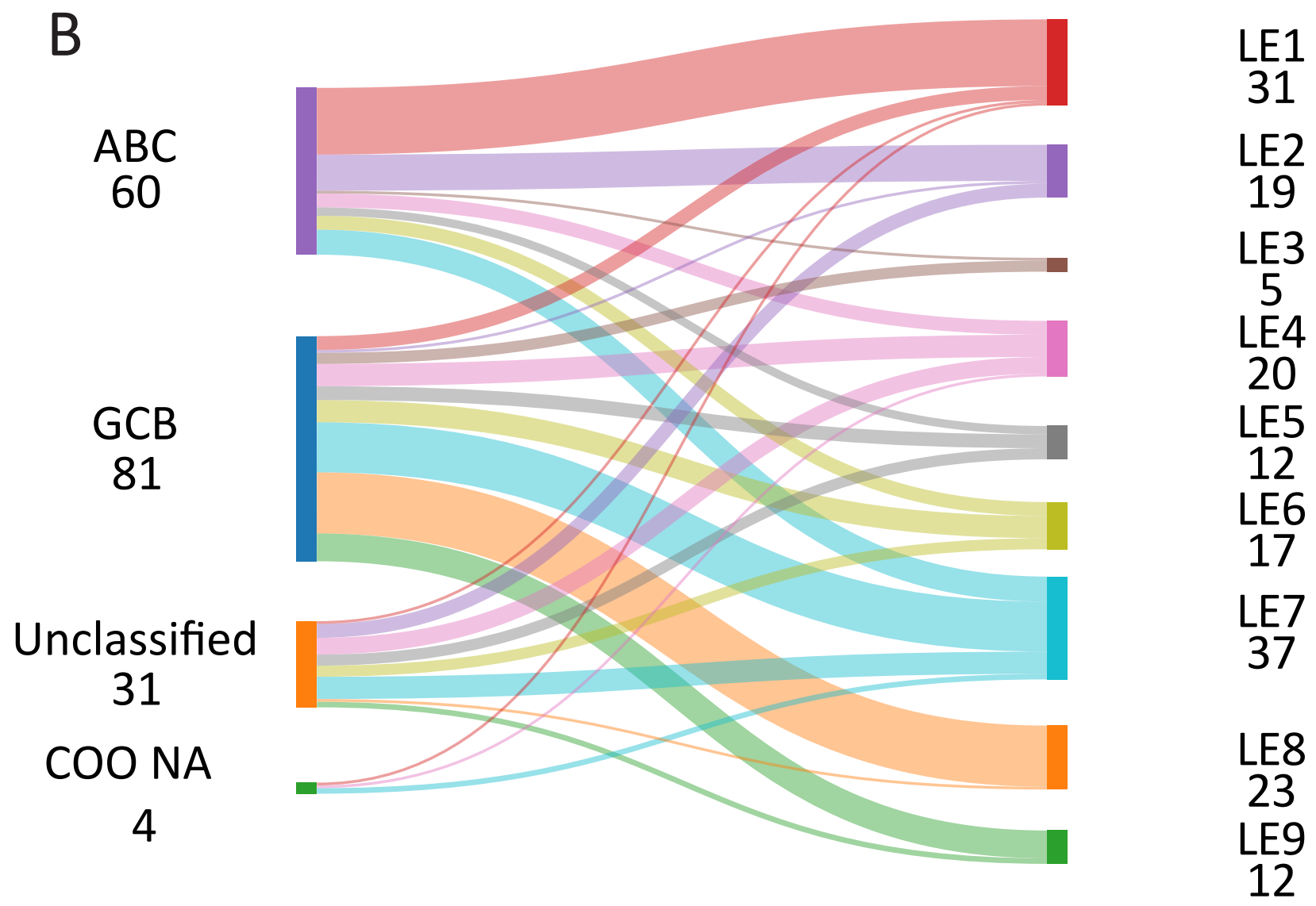

ABC
60
GCB
81
Unclassified
31
COO NA
4
LE1
31
LE2
19
LE3
5
LE4
20
LE5
12
LE6
17
LE7
37
LE8
23
LE9
12
