# Supplementary material for: Dissecting diffuse large B‐cell lymphoma heterogeneity: New insights from a state‐specific molecular characterisation
Source: Clin Transl Med. 2026 Aug 19;16(8):e70757. doi: 10.1002/ctm2.70757 (PMC13487464; doi:10.1002/ctm2.70757)
Supplement: Supplementary file 2 [file CTM2-16-e70757-s003.pdf]

Figure S2

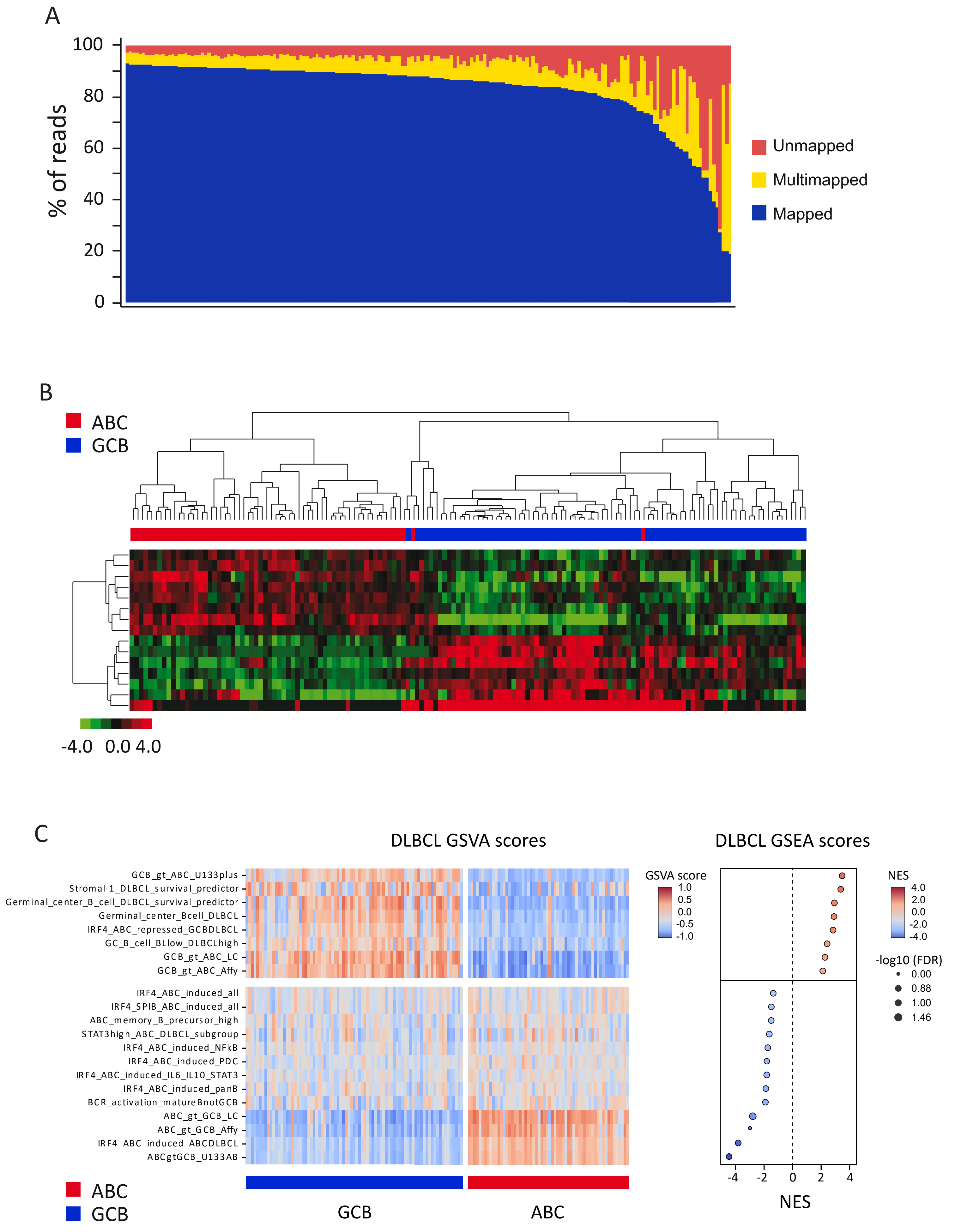

A
100
80
60
40
20
0
% of reads
Unmapped
Multimapped
Mapped
B
ABC
GCB
-4.0
0.0
4.0
C
DLBCL GSVA scores
DLBCL GSEA scores
GCB_gt_ABC_U133plus
Stromal-1_DLBCL_survival_predictor
Germinal_center_B_cell_DLBCL_survival_predictor
Germinal_center_Bcell_DLBCL
IRF4_ABC_repressed_GCBDLBCL
GC_B_cell_BLlow_DLBCLhigh
GCB_gt_ABC_LC
GCB_gt_ABC_Affy
IRF4_ABC_induced_all
IRF4_SPIB_ABC_induced_all
ABC_memory_B_precursor_high
STAT3high_ABC_DLBCL_subgroup
IRF4_ABC_induced_NFkB
IRF4_ABC_induced_PDC
IRF4_ABC_induced_IL6_IL10_STAT3
IRF4_ABC_induced_panB
BCR_activation_matureBnotGCB
ABC_gt_GCB_LC
ABC_gt_GCB_Affy
IRF4_ABC_induced_ABCDLBCL
ABCgtGCB_U133AB
GSVA score
1.0
0.5
0.0
-0.5
-1.0
NES
4.0
2.0
0.0
-2.0
-4.0
-log10 (FDR)
0.00
0.88
1.00
1.46
-4
-2
0
2
4
NES
ABC
GCB
GCB
ABC
