# Supplementary material for: Dissecting diffuse large B‐cell lymphoma heterogeneity: New insights from a state‐specific molecular characterisation
Source: Clin Transl Med. 2026 Aug 19;16(8):e70757. doi: 10.1002/ctm2.70757 (PMC13487464; doi:10.1002/ctm2.70757)

Figure S10

A

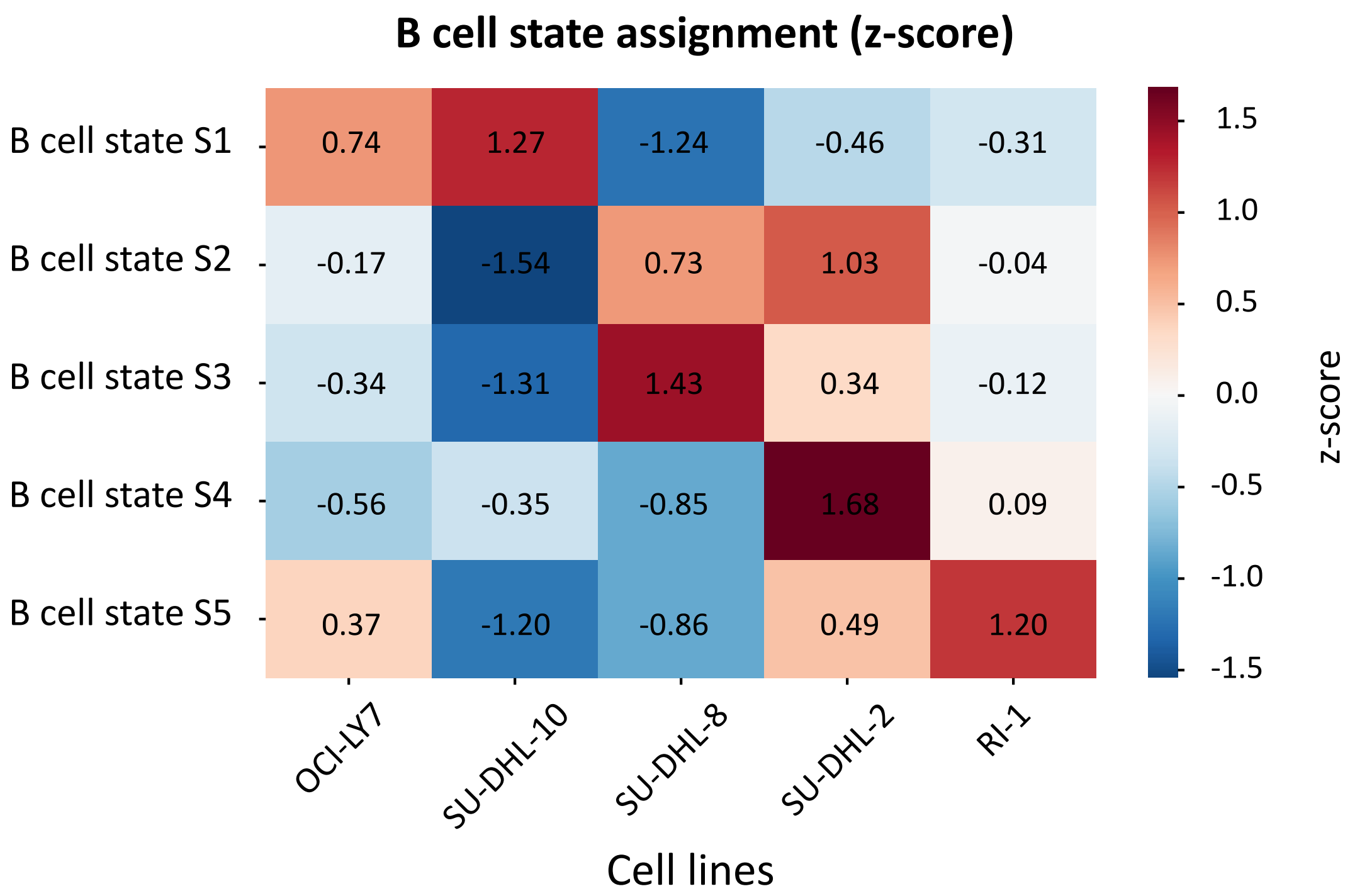

B

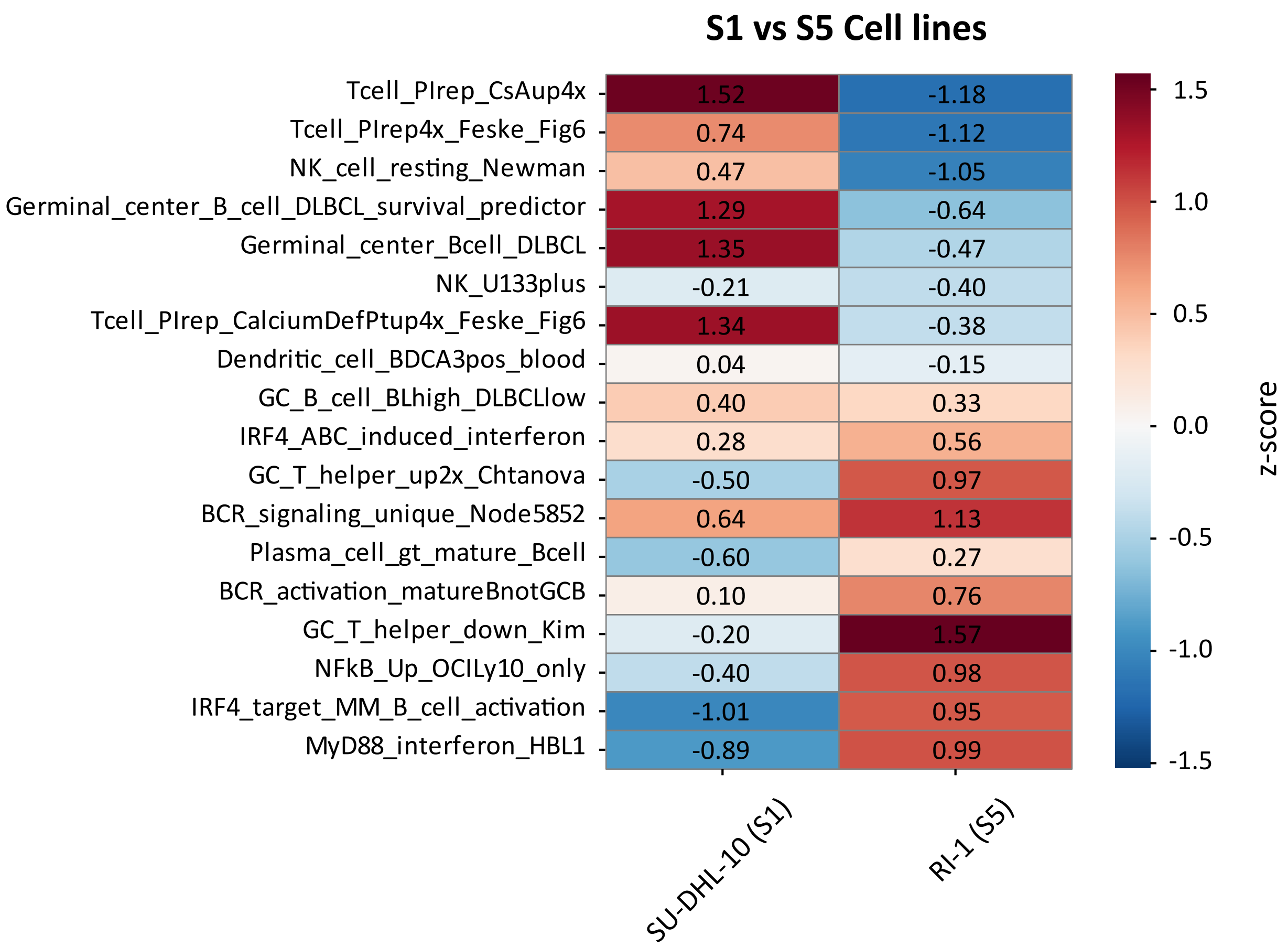

Supplement: Supplementary file 3 [file CTM2-16-e70757-s004.pdf]
