# Supplementary material for: Dissecting diffuse large B‐cell lymphoma heterogeneity: New insights from a state‐specific molecular characterisation
Source: Clin Transl Med. 2026 Aug 19;16(8):e70757. doi: 10.1002/ctm2.70757 (PMC13487464; doi:10.1002/ctm2.70757)
Supplement: Supplementary file 4 [file CTM2-16-e70757-s005.pdf]

Figure S8

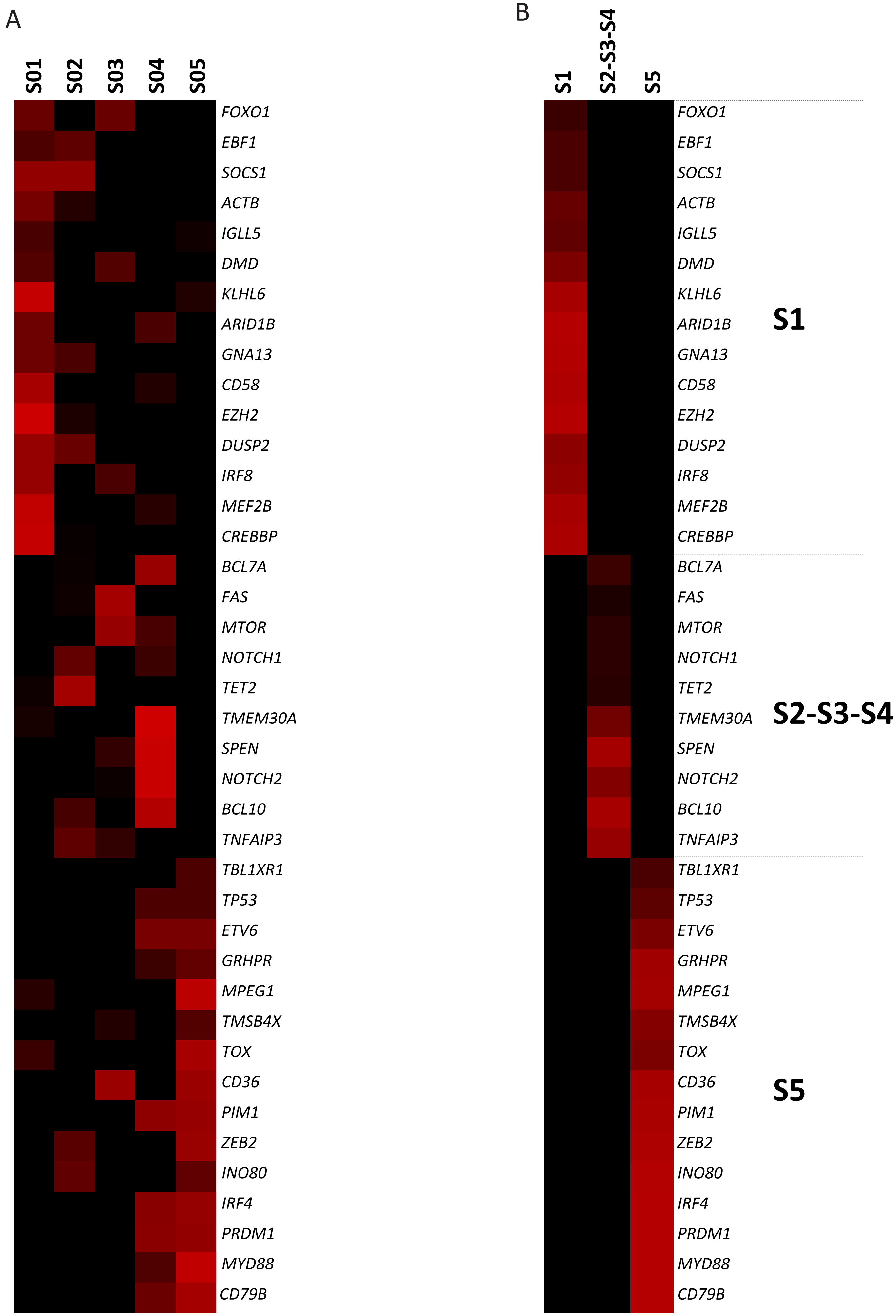
A
S01
S02
S03
S04
S05
FOXO1
EBF1
SOCS1
ACTB
IGLL5
DMD
KLHL6
ARID1B
GNA13
CD58
EZH2
DUSP2
IRF8
MEF2B
CREBBP
BCL7A
FAS
MTOR
NOTCH1
TET2
TMEM30A
SPEN
NOTCH2
BCL10
TNFAIP3
TBL1XR1
TP53
ETV6
GRHPR
MPEG1
TMSB4X
TOX
CD36
PIM1
ZEB2
INO80
IRF4
PRDM1
MYD88
CD79B
B
S1
S2-S3-S4
S5
S1
S2-S3-S4
S5
