# Supplementary material for: Dissecting diffuse large B‐cell lymphoma heterogeneity: New insights from a state‐specific molecular characterisation
Source: Clin Transl Med. 2026 Aug 19;16(8):e70757. doi: 10.1002/ctm2.70757 (PMC13487464; doi:10.1002/ctm2.70757)
Supplement: Supplementary file 5 [file CTM2-16-e70757-s006.pdf]

Figure S3

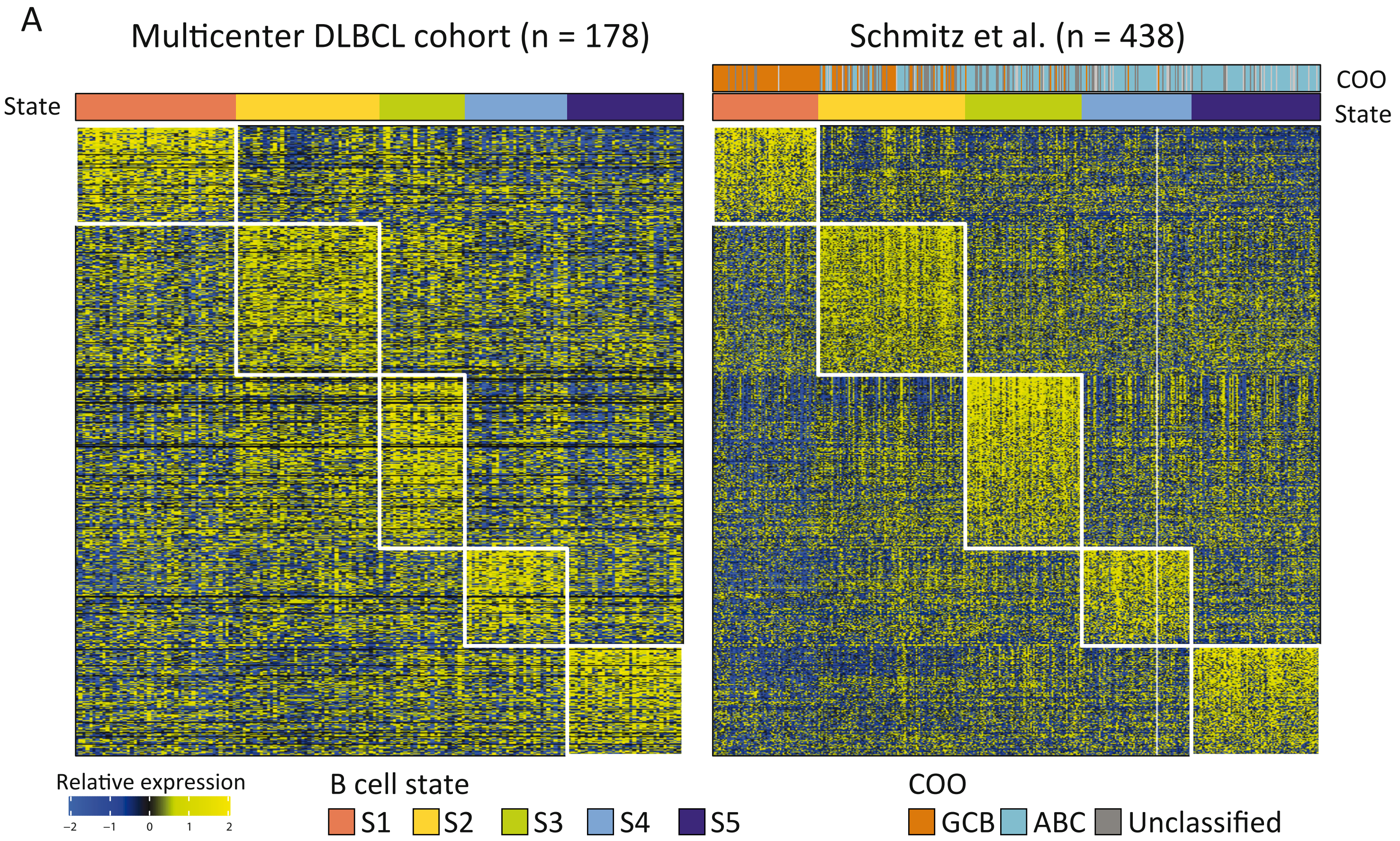
A
Multicenter DLBCL cohort (n = 178)
Schmitz et al. (n = 438)
State
COO
State
Relative expression
−2
−1
0
1
2
B cell state
S1
S2
S3
S4
S5
COO
GCB
ABC
Unclassified
