# Supplementary material for: Dissecting diffuse large B‐cell lymphoma heterogeneity: New insights from a state‐specific molecular characterisation
Source: Clin Transl Med. 2026 Aug 19;16(8):e70757. doi: 10.1002/ctm2.70757 (PMC13487464; doi:10.1002/ctm2.70757)
Supplement: Supplementary file 6 [file CTM2-16-e70757-s007.pdf]

Figure S1

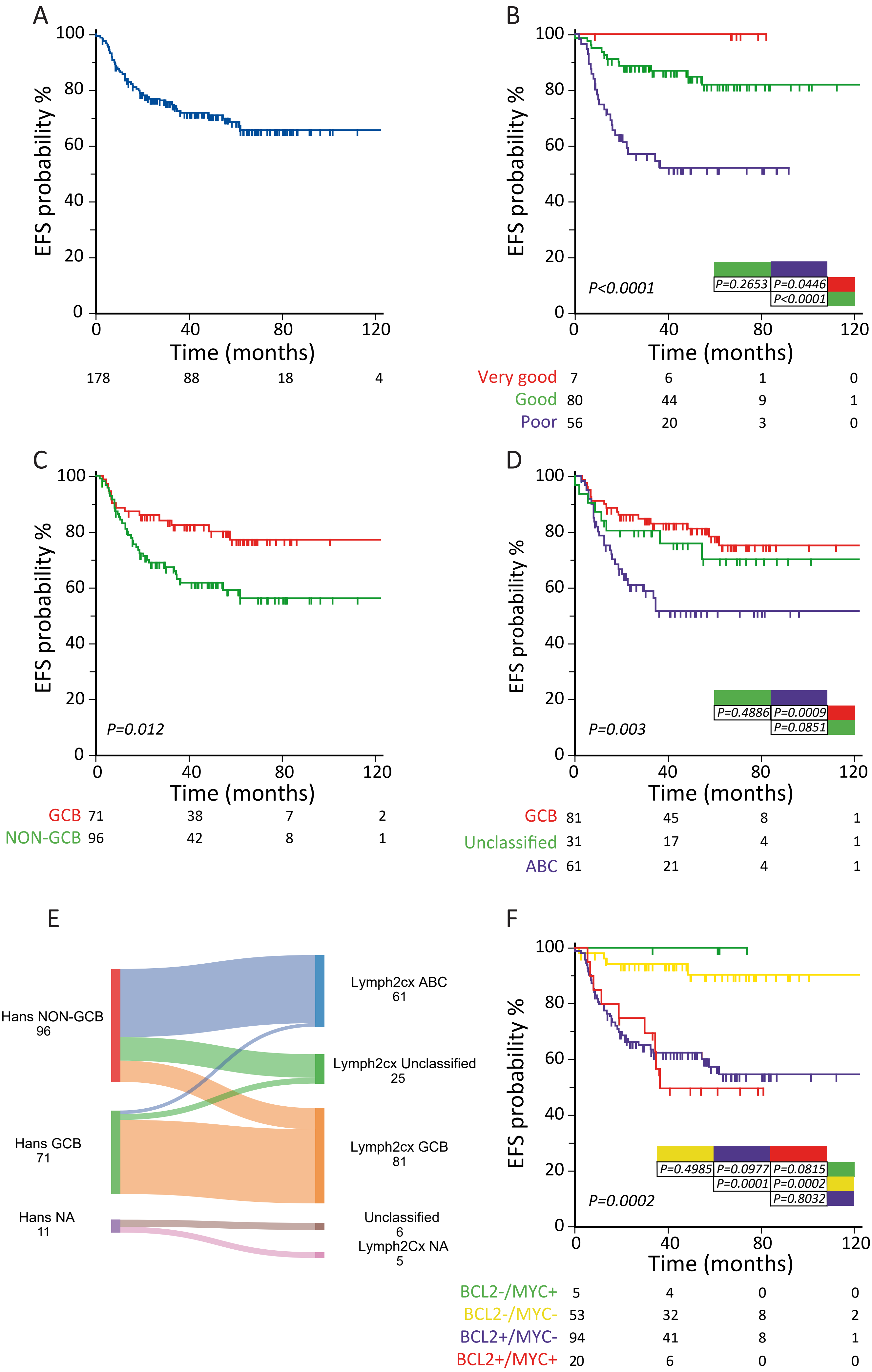

A
EFS probability %
100
80
60
40
20
0
0
40
80
120
Time (months)
178
88
18
4
B
EFS probability %
Time (months)
P<0.0001
P=0.2653
P=0.0446
P<0.0001
Very good 7 6 1 0
Good 80 44 9 1
Poor 56 20 3 0
C
EFS probability %
Time (months)
P=0.012
GCB 71 38 7 2
NON-GCB 96 42 8 1
D
EFS probability %
Time (months)
P=0.003
P=0.4886
P=0.0009
P=0.0851
GCB 81 45 8 1
Unclassified 31 17 4 1
ABC 61 21 4 1
E
Hans NON-GCB
96
Hans GCB
71
Hans NA
11
Lymph2cx ABC
61
Lymph2cx Unclassified
25
Lymph2cx GCB
81
Unclassified
6
Lymph2Cx NA
5
F
EFS probability %
Time (months)
P=0.0002
P=0.4985
P=0.0977
P=0.0815
P=0.0001
P=0.0002
P=0.8032
BCL2-/MYC+ 5 4 0 0
BCL2-/MYC- 53 32 8 2
BCL2+/MYC- 94 41 8 1
BCL2+/MYC+ 20 6 0 0
