# Supplementary material for: Dissecting diffuse large B‐cell lymphoma heterogeneity: New insights from a state‐specific molecular characterisation
Source: Clin Transl Med. 2026 Aug 19;16(8):e70757. doi: 10.1002/ctm2.70757 (PMC13487464; doi:10.1002/ctm2.70757)
Supplement: Supplementary file 7 [file CTM2-16-e70757-s008.pdf]

Figure S9

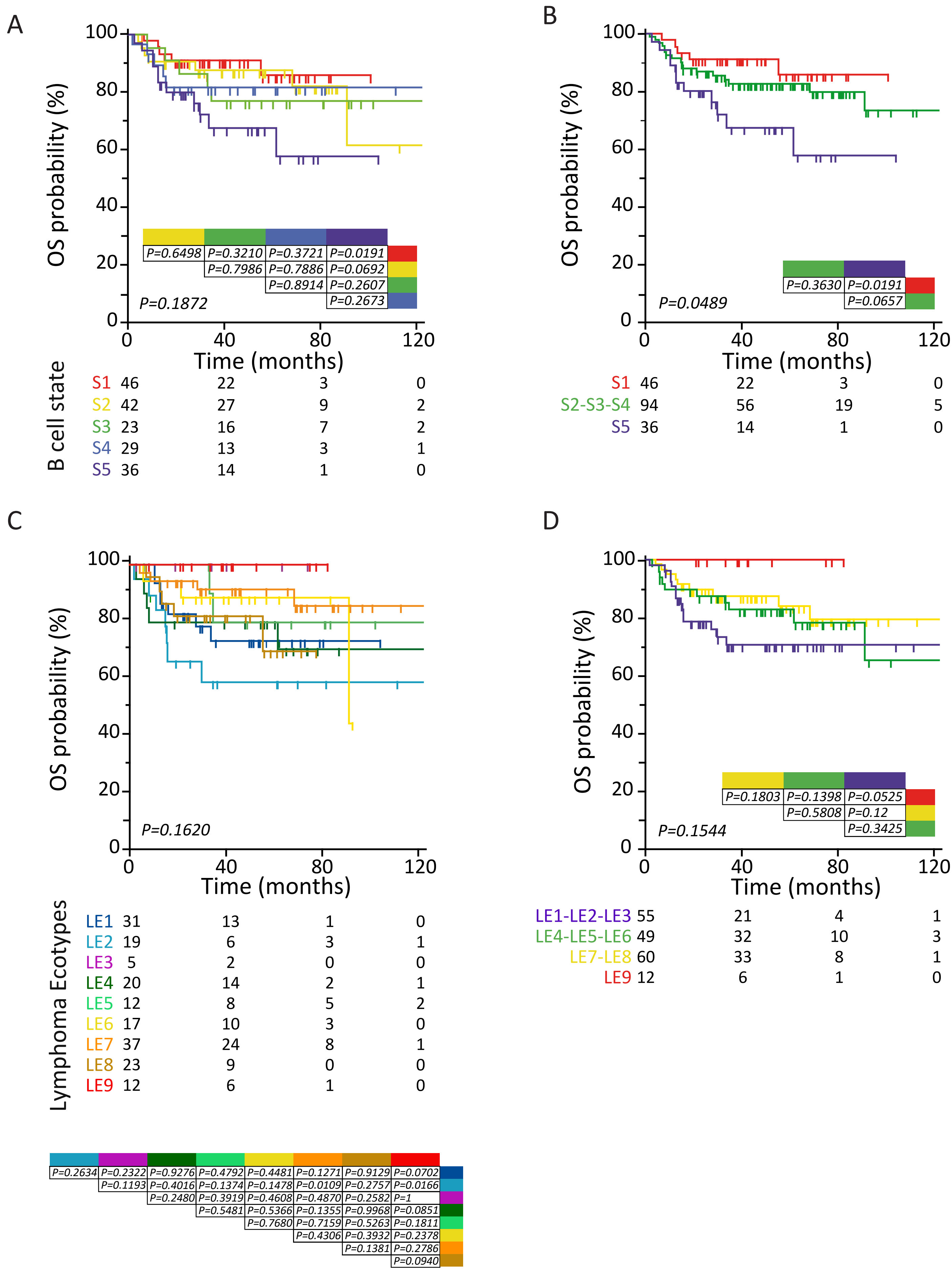
A
OS probability (%)
Time (months)
P=0.6498
P=0.3210
P=0.3721
P=0.0191
P=0.7986
P=0.7886
P=0.0692
P=0.8914
P=0.2607
P=0.2673
P=0.1872
B cell state
S1 46 22 3 0
S2 42 27 9 2
S3 23 16 7 2
S4 29 13 3 1
S5 36 14 1 0
B
OS probability (%)
Time (months)
P=0.3630
P=0.0191
P=0.0657
P=0.0489
S1 46 22 3 0
S2-S3-S4 94 56 19 5
S5 36 14 1 0
C
OS probability (%)
Time (months)
P=0.1620
Lymphoma Ecotypes
LE1 31 13 1 0
LE2 19 6 3 1
LE3 5 2 0 0
LE4 20 14 2 1
LE5 12 8 5 2
LE6 17 10 3 0
LE7 37 24 8 1
LE8 23 9 0 0
LE9 12 6 1 0
P=0.2634 P=0.2322 P=0.9276 P=0.4792 P=0.4481 P=0.1271 P=0.9129 P=0.0702
P=0.1193 P=0.4016 P=0.1374 P=0.1478 P=0.0109 P=0.2757 P=0.0166
P=0.2480 P=0.3919 P=0.4608 P=0.4870 P=0.2582 P=1
P=0.5481 P=0.5366 P=0.1355 P=0.9968 P=0.0851
P=0.7680 P=0.7159 P=0.5263 P=0.1811
P=0.4306 P=0.3932 P=0.2378
P=0.1381 P=0.2786
P=0.0940
D
OS probability (%)
Time (months)
P=0.1803
P=0.1398
P=0.0525
P=0.5808
P=0.12
P=0.3425
P=0.1544
LE1-LE2-LE3 55 21 4 1
LE4-LE5-LE6 49 32 10 3
LE7-LE8 60 33 8 1
LE9 12 6 1 0
