# Supplementary material for: Dissecting diffuse large B‐cell lymphoma heterogeneity: New insights from a state‐specific molecular characterisation
Source: Clin Transl Med. 2026 Aug 19;16(8):e70757. doi: 10.1002/ctm2.70757 (PMC13487464; doi:10.1002/ctm2.70757)
Supplement: Supplementary file 8 [file CTM2-16-e70757-s011.pdf]

Figure S5

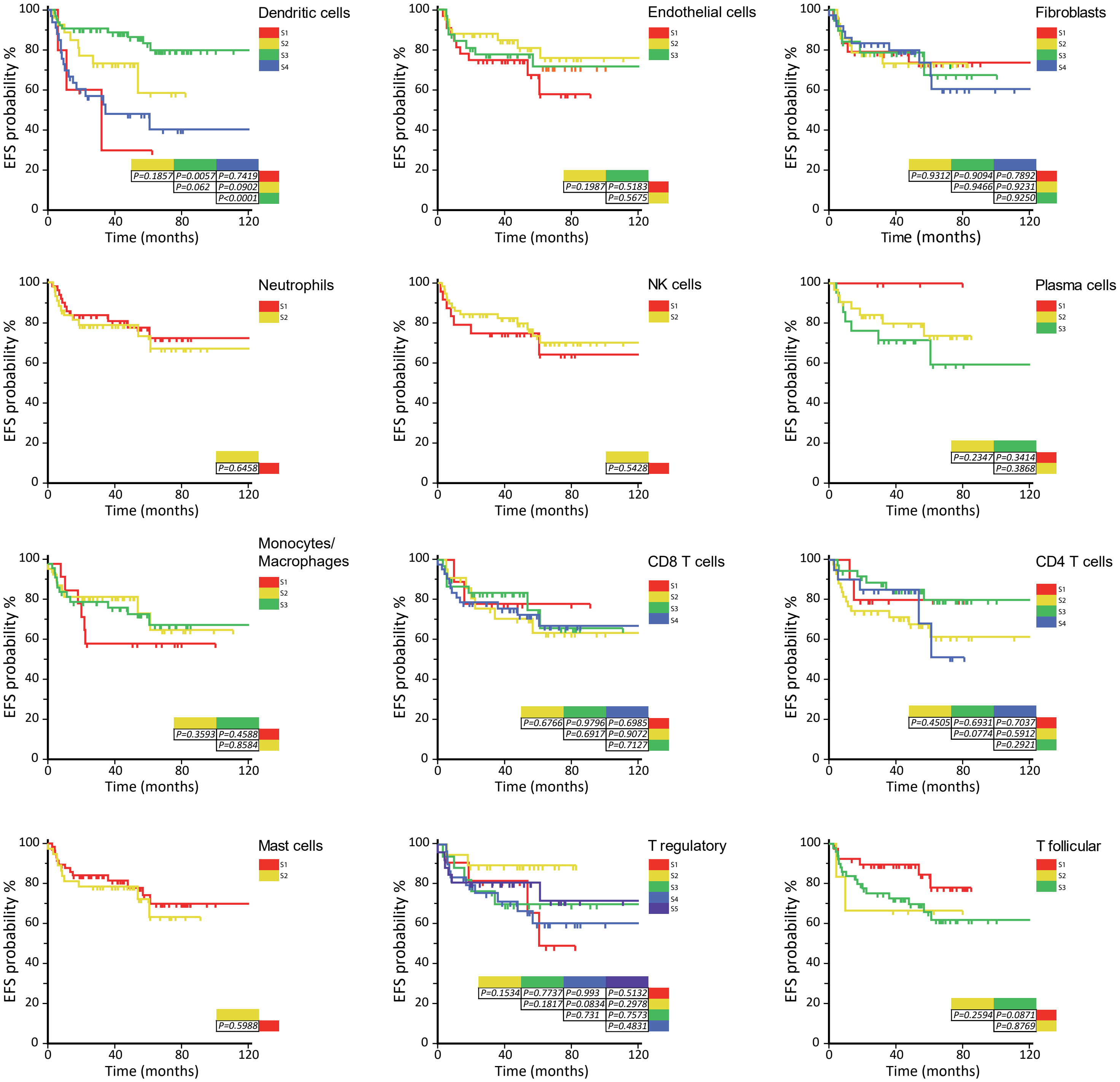
EFS probability %
Time (months)
Dendritic cells
S1
S2
S3
S4
P=0.1857
P=0.0057
P=0.7419
P=0.062
P=0.0902
P<0.0001
Endothelial cells
S1
S2
S3
P=0.1987
P=0.5183
P=0.5675
Fibroblasts
S1
S2
S3
S4
P=0.9312
P=0.9094
P=0.7892
P=0.9466
P=0.9231
P=0.9250
Neutrophils
S1
S2
P=0.6458
NK cells
S1
S2
P=0.5428
Plasma cells
S1
S2
S3
P=0.2347
P=0.3414
P=0.3868
Monocytes/ Macrophages
S1
S2
S3
P=0.3593
P=0.4588
P=0.8584
CD8 T cells
S1
S2
S3
S4
P=0.6766
P=0.9796
P=0.6985
P=0.6917
P=0.9072
P=0.7127
CD4 T cells
S1
S2
S3
S4
P=0.4505
P=0.6931
P=0.7037
P=0.0774
P=0.5912
P=0.2921
Mast cells
S1
S2
P=0.5988
T regulatory
S1
S2
S3
S4
S5
P=0.1534
P=0.7737
P=0.993
P=0.5132
P=0.1817
P=0.0834
P=0.2978
P=0.731
P=0.7573
P=0.4831
T follicular
S1
S2
S3
P=0.2594
P=0.0871
P=0.8769
