# Supplementary material for: Dissecting diffuse large B‐cell lymphoma heterogeneity: New insights from a state‐specific molecular characterisation
Source: Clin Transl Med. 2026 Aug 19;16(8):e70757. doi: 10.1002/ctm2.70757 (PMC13487464; doi:10.1002/ctm2.70757)
Supplement: Supplementary file 9 [file CTM2-16-e70757-s013.pdf]

Figure S6

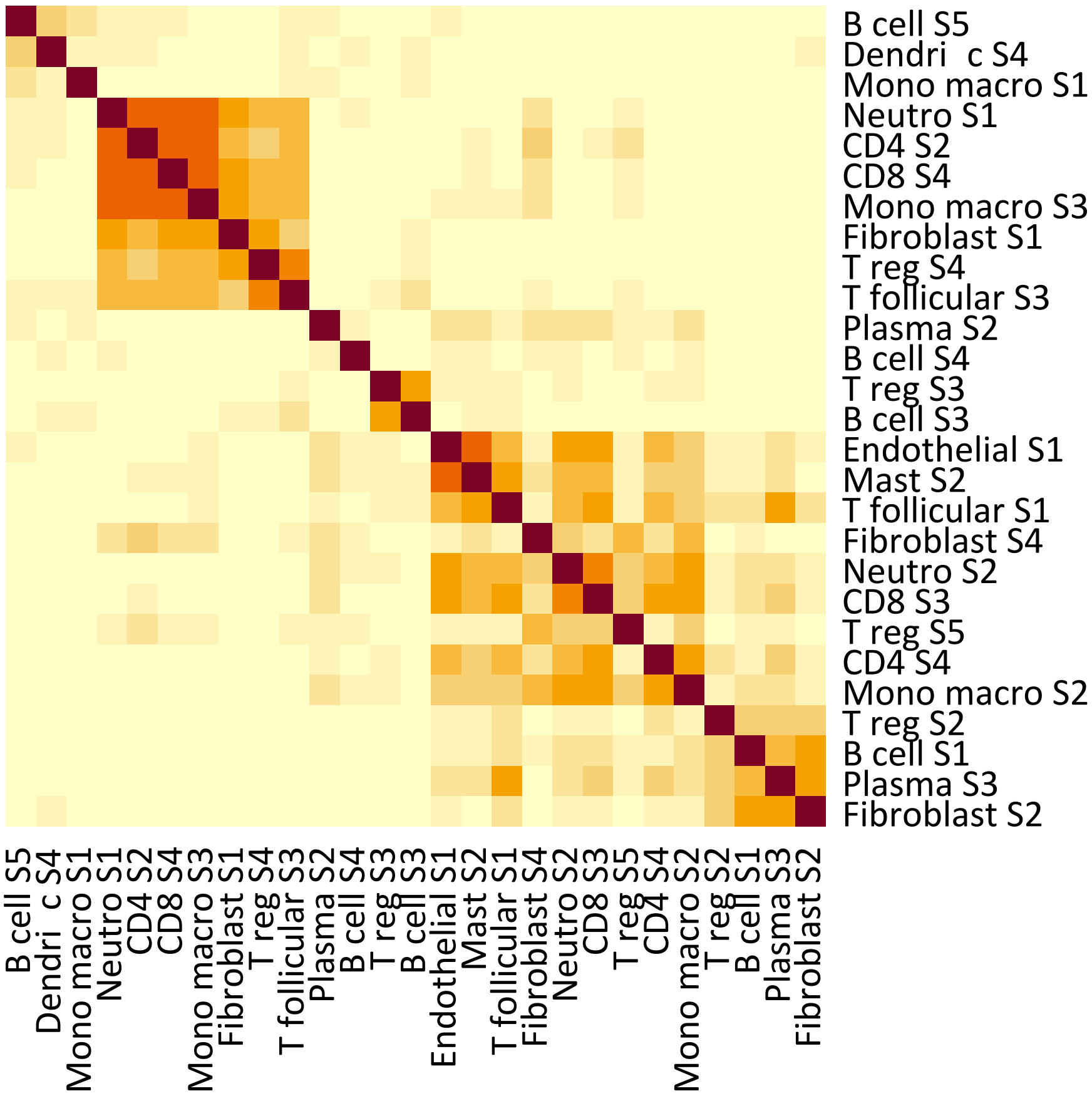
B cell S5
Dendri c S4
Mono macro S1
Neutro S1
CD4 S2
CD8 S4
Mono macro S3
Fibroblast S1
T reg S4
T follicular S3
Plasma S2
B cell S4
T reg S3
B cell S3
Endothelial S1
Mast S2
T follicular S1
Fibroblast S4
Neutro S2
CD8 S3
T reg S5
CD4 S4
Mono macro S2
T reg S2
B cell S1
Plasma S3
Fibroblast S2
