# Supplementary material for: Dissecting diffuse large B‐cell lymphoma heterogeneity: New insights from a state‐specific molecular characterisation
Source: Clin Transl Med. 2026 Aug 19;16(8):e70757. doi: 10.1002/ctm2.70757 (PMC13487464; doi:10.1002/ctm2.70757)
Supplement: Supplementary file 11 [file CTM2-16-e70757-s009.docx]

# Dissecting diffuse large B cell lymphoma heterogeneity: new insights from a state-specific molecular characterization

**Running Title:** Real-life state-specific DLBCL characterization

Robel Papotti^1A^, Filippo Vit^1A^, Tamara Bittolo^1^, Federico Pozzo^1^, Stefania Bettelli^2^, Samantha Pozzi^3^, Luca Braglia^3^, Elisa Forti^2^, Arianna Di Napoli^4^, M. Christina Cox^5^, Tamar Tadmor^6^, Pellegrino Musto^7^, Leonardo Flenghi^8^, Martina Quintini^8^, Giovanna R. Mansueto^9^, Sara Galimberti^10^, Valentina Donati^11^, Michele Spina^12^, Alberto Zamò^13^, Andreas Rosenwald^13^, Riccardo Bomben^1B^, Stefano Sacchi^3B^ and Valter Gattei^1B^

^A^Equally contributed to this work

^B^Equally contributed as senior authors

^1^Clinical and Experimental Onco-Hematology Unit, Centro di Riferimento Oncologico di Aviano (CRO), IRCCS, Italy

^2^Molecular Pathology and Predictive Medicine Unit, Modena University Hospital, Modena, Italy

^3^Oncohematology and Osteoncology Unit, Modena University Hospital, Modena, Italy

^4^Pathology Unit, Sapienza University Sant'Andrea Hospital, Rome, Italy

^5^Fondazione Policlinico Tor Vergata, University Hospital of Rome, Italy

^6^Hematology Unit, Bnai Zion Medical Center, Haifa, Israel

^7^Aldo Moro University School of Medicine and Unit of Hematology and Stem Cell Transplantation, AOU Consorziale Policlinico, Bari, Italy

^8^Department of Emergency and Organ Transplantation, Azienda Ospedaliera di Perugia, Italy

^9^Pathology Unit, IRCCS-CROB of Rionero in Vulture, Italy

^10^Hematology Unit, Azienda Ospedaliero-Universitaria Pisana, Pisa, Italy

^11^Pathology Unit, Azienda Ospedaliero-Universitaria Pisana, Pisa, Italy

^12^Oncologia medica e dei tumori immuno-correlati, Centro di Riferimento Oncologico di Aviano (CRO), IRCCS, Italy

^13^Pathology Unit, Julius-Maximilians-Universität, Würzburg, Germany

**Supplemental Information**

# Dissecting diffuse large B cell lymphoma heterogeneity: new insights from a state-specific molecular characterization

**Supplemental Figures:**

- **Figure S1. Clinical characteristics and survival curves of the DLBCL cohort.**
- **Figure S2. Sequencing statistics and RNA-Seq analysis.**
- **Figure S3. EcoTyper analysis comparison.**
- **Figure S4. Sankey diagram distributions.**
- **Figure S5. Tumor microenvironment cell states associated survival curves.**
- **Figure S6. Cell states co-occurrence analysis.**
- **Figure S7. Lymphgen analysis.**
- **Figure S8. Mutational characterization of B cell states.**
- **Figure S9. Overall survival according to B cell states and Lymphoma Ecotypes classifications.**
- **Figure S10. Transcriptomic enrichment profiling of B cell state signatures in DLBCL cell lines.**

**Supplemental Tables:**

- **Table S1. Antibodies and clones list used for Immunohistochemistry.**
- **Table S2. Genomic regions targeted by the sequencing panel (hg19).**
- **Table S3. Cell states, Lymphoma EcoTypes and Lymphgen subtype identified in DLBCL cohort.**
- **Table S4. Clinical correlation in the context of the top 15 mutated genes.**

# Material and Methods

## DLBCL cohorts

This study, generated in the framework of an multicenter institutional working group on diffuse large B cell lymphoma (DLBCL), in collaboration with Modena Cancer Center, S. Andrea Rome Hospital, Hematology Unit of Pisa University Hospital, Hematology Unit of Rionero in Vulture Referral Cancer Center, Hematology Unit of Perugia Hospital, and Bnai Zion Haifa Medical Center (Israel), is a retrospective/multicenter analysis on 204 DLBCL patients treated with R-CHOP or R-CHOP-like regimens in the current clinical practice (January/2008-April/2019). The selection of cases was driven by the availability of adequate FFPE tissue for molecular analysis, and clinical data.

## RNA sequencing

Preparation of sequencing libraries for RNA sequencing (RNA-Seq) was performed using 150 ng of total extracted RNA from formalin fixed and paraffin embedded FFPE samples with the RNA HyperPrep with RiboErase (HMR) kit (Roche Sequencing Solutions). Briefly, to enable higher sequencing depth of mRNA and better detect transcripts, a rRNA depletion method was utilized to eliminate both cytoplasmic (5S, 5.8S, 18S, and 28S) and mitochondrial (12S and 16S) rRNA species. Subsequently, the RNA:DNA hybrids were degraded with RNAse-H, and processed accordingly to the manufacturer’s instructions (Roche).

Libraries quality control was assessed using Agilent 2200 TapeStation (Agilent Technologies, Inc.), and quantitative real time PCR (qRT-PCR). Eventually, libraries were pooled together in equimolar ratio in two separate pools. Pools were sequenced on S4 flow-cells using a NovaSeq6000 platform (Illumina) 2x150 cycles.

## RNA-Seq analysis

Raw counts were subjected to a filtering step using an in-house script, requiring at least half of the samples to have a total count exceeding 10 for a specific gene. Differentially expressed genes were identified with glmQLFit/glmQLFTest functions in edgeR^1^ which implement quasi-likelihood (QL) methods. Genes with a log fold change (FC) higher than 1 with a false discovery rate (FDR) lower than 1×10^-4^ were considered differentially expressed between the groups. Using as a cutoff value an adjusted P<0.01 and a fold change (FC)>2.

To confirm Lymph2cx classification using RNA-Seq data, among the 20 genes used by Lymph2cx algorithm^2^ we extrapolated only the counts of the 15 specific genes employed for the cell of origin (COO) classification with the exclusion of the 5 house keeping genes. Raw data were normalized using the Trimmed mean of M-values (TMM) log2 transformation. Data were then clustered with hclust (row clustering by Pearson correlation, columns clustering by Spearman correlation using Ward's minimum variance method).

## Targeted sequencing

Analysis of cBioPortal database (http://www.cbioportal.org/) and COSMIC database (https://cancer.sanger.ac.uk/cosmic) filtering for studies related to "Lymphoid" and "diffuse large B cell lymphoma (DLBCL)" allow the selection of ~1.250 DLBCL samples analyzed through whole genome or exon sequencing. Known driver genes that were currently mutated in >3% of B cell tumors were selected for the CAPP-seq panel, resulting in a final panel of 169 genes (Table S2).

Genomic DNA was co-extracted alongside total RNA using the AllPrep DNA/RNA Kit (Qiagen, Hilden, Germany) from FFPE tissue samples according to the manufacturer’s instructions. Library preparation was performed starting from 100 ng of DNA, incorporating an initial enzymatic DNA polishing phase to repair and mitigate FFPE-induced sequence artifacts, and following the Kapa HyperPrep Kit (Roche Sequencing Solutions) instructions. Libraries quality control was assessed using Agilent 2200 TapeStation (Agilent Technologies, Inc.), and quantitative real time PCR (qRT-PCR). Eventually, libraries were pooled together in equimolar ratio and sequenced on S4 flow-cell using a NovaSeq6000 platform (Illumina) 2x150 cycles.FASTQ reads were deduped and aligned to the human genome hg38 version using FastUniq, and BWA software. Single nucleotide variations and indels were called using LoFreq^3^ and annotated with Annovar.^4^ Variants annotated as SNPs (dbSNP138), intronic, and synonymous were filtered out. Only mutation with a variant allele frequency (VAF) >5% and with a REVEL (rare exome variant ensemble learner) score >0.4 were used for subsequent analysis. Mutation enrichment per cell state were normalized using StandardScaler function of scikit-learn package.

DLBCL samples were classified using the LymphGen algorithm (<https://llmpp.nih.gov/lymphgen>), using somatic mutations to define genetic subtypes . ^5^ High-confidence variant call data obtained through CAPP-Seq analysis were used as input, following the standard LymphGen classification framework. Each sample was assigned to one of the five molecular subtypes (MCD, BN2, N1, EZB, ST2) based on the comparison of confidence values or categorized as "Other" if it lacked defining genomic features.

# Results

## Cell of origin classification

Immunohistochemistry (IHC), based on the Hans' algorithm,^6^ identified 71 (42.5%) cases as germinal center like (GCB), and 96 (57.5%) as non-GCB. As reported in Figure S1C, GCB patients experienced longer event free survival (EFS) (mean 92.7 months) than non-GCB patients (mean 90.4 months; *P=0.0111*; Figure S1C). A separate assessment using the Lymph2Cx assay delineated 81 (46.8%) cases as GCB, 61 (35.3%) as activated B cell like (ABC), and 31 (17.9%) as Unclassified. Notably, 5 samples out 178 (2.8%) did not meet the quality requirements for the Lymph2Cx assay. Again, GCB patients showed longer EFS (mean 90.9 months) than ABC patients (mean 82.5 months; *P=0.0008*; Figure S1D). As expected, Unclassified patients displayed an intermediate behavior between GCB and ABC classification^2^. Comparison between the two COO classification methods revealed that 3 cases initially classified as GCB according to the Hans' algorithm were categorized as ABC using the Lymph2Cx assay, while 18 non-GCB patients demonstrated GCB characteristics with Lymph2Cx (Figure S1E). Upon exclusion of the 31 Unclassified cases (5 GCB, 20 non-GCB, and 6 with no IHC) a comparison value of 0.71 was calculated for the k-statistic, indicating good concordance between the IHC and Lymph2Cx classification.

## BCL2 and MYC expression

Analysis of BCL2 expression identified 58 cases (33%) as BCL2-, and 116 (67%) as BCL2+, while MYC expression showed 148 (85%) cases MYC-, and 25 (15%) cases MYC+ (Table 1). Combining the simultaneous expression of BCL2, and MYC: i) 53 cases resulted BCL2-/MYC- (31%; double-negative), ii) 5 cases BCL2-/MYC+ (3%), iii) 94 cases BCL+/MYC- (55%), and iv) 20 cases BCL2+/MYC+ (11%), also known as double expressor (Table 1). Statistical analysis showed a 5-year EFS of 91% for BCL2-/MYC-, 54% for BCL2+/MYC-, and of 51% for BCL2+/MYC+. The comparable poor outcomes of the latter two groups suggest that, in this cohort, BCL2 positivity drove the adverse prognosis irrespective of MYC (BCL2-/MYC- vs BCL2+/MYC- *P=0.0001*; BCL2-/MYC- vs BCL2+/MYC+ *P=0.0002*; Figure S1F).

## RNA sequencing

In the context of the 204 patients, 192 cases presented adequate material for total RNA sequencing investigation. Eventually, 186/192 (97%) cases generated a successful RNA-Seq libraries, and were subsequently sequenced. Given the FFPE nature of the samples, RNA quality was monitored using the DV200 metric to ensure library complexity. To control for potential technical biases arising from different sequencing runs, batch information was explicitly modeled as a covariate in the generalized linear model design during differential expression analysis. This approach allowed for the statistical adjustment of batch effects while preserving biological variance. Despite the use of RNA from FFPE samples, libraries were globally well balanced, showing an average of 2.26×10^8^ number of reads, and samples presented a consistent percentage of uniquely mapped reads with a median percentage of 82.43% (Figure S2A).

## Genetic mutations and profiles

By feeding LymphGen tool with the genetic lesions detected in FFPE samples, despite the absence of FISH data, 79 (54%) out of 146 patients were assigned to a molecular subtype with a median confidence score exceeding 87%, while 67 (46%) samples were classified as “Other”. Specifically, 18 (12%) patients were classified as MCD, 20 (14%) as ST2, 16 (11%) as EZB, 3 (2%) as N1, and 22 (15%) as BN2 (Figure S7A). Importantly, potential A53 cases were grouped within the “Other” category due to the lack of copy number variation data, as *TP53* mutational status alone is not sufficient for classification. Excluding patients in the “Other” subgroup, 69 of 79 cases with available clinical data were analyzed. Among these, the MCD (characterized by *CD79B* and *MYD88* mutations) and BN2 (characterized by *NOTCH2* mutations) subgroups demonstrated the poorest clinical outcomes (Figure S7B).

**Acknowledgment:** The present study is supported in part by: Fondazione Italiana Linfomi (FIL) Young Researcher Grant GR2020, Fondazione Giulia Maramotti e Fondazione GRADE Onlus; PNRR-MCNT2-2023-12378037, Italian Ministry of Health (Ricerca Corrente); Associazione Italiana contro le Leucemie, linfomi e mielomi (AIL), Venezia Section, Italy; Associazione Mantovana per la Ricerca sul Cancro (A.MA.RI.CA).

**References**

1.	Robinson MD, McCarthy DJ, Smyth GK. edgeR: a Bioconductor package for differential expression analysis of digital gene expression data. *Bioinformatics*. 2010;26(1):139-140. doi:10.1093/bioinformatics/btp616

2.	Scott DW, Wright GW, Williams PM, et al. Determining cell-of-origin subtypes of diffuse large B-cell lymphoma using gene expression in formalin-fixed paraffin-embedded tissue. *Blood*. 2014;123(8):1214-1217. doi:10.1182/blood-2013-11-536433

3.	Wilm A, Aw PPK, Bertrand D, et al. LoFreq: a sequence-quality aware, ultra-sensitive variant caller for uncovering cell-population heterogeneity from high-throughput sequencing datasets. *Nucleic Acids Res*. 2012;40(22):11189-11201. doi:10.1093/nar/gks918

4.	Wang K, Li M, Hakonarson H. ANNOVAR: functional annotation of genetic variants from high-throughput sequencing data. *Nucleic Acids Res*. 2010;38(16):e164. doi:10.1093/nar/gkq603

5.	Wright GW, Huang DW, Phelan JD, et al. A Probabilistic Classification Tool for Genetic Subtypes of Diffuse Large B Cell Lymphoma with Therapeutic Implications. *Cancer Cell*. 2020;37(4):551-568.e14. doi:10.1016/j.ccell.2020.03.015

6.	Hans CP, Weisenburger DD, Greiner TC, et al. Confirmation of the molecular classification of diffuse large B-cell lymphoma by immunohistochemistry using a tissue microarray. *Blood*. 2004;103(1):275-282. doi:10.1182/blood-2003-05-1545

**Figure Legends**

**Figure S1. Clinical characteristics and survival curves of the DLBCL cohort.** A) Kaplan-Meier curve showing event free survival (EFS) probabilities of 178 DLBCL patients. Median follow-up was of 42.2 months (95% CI 35.4-49.4 months) with 3-years and 5-years EFS of 72.3%, and 65.8% respectively A total of 52 events were registered, 33 of which were deaths. B) Kaplan-Meier curves comparing EFS probabilities of 143 DLBCL patients according to R-IPI classification. The graph shows 7 Very good (red line), 80 Good (green line) and 56 Poor (purple line) DLBCL cases. C) Kaplan-Meier curves comparing EFS probabilities of 167 DLBCL patients according to Cell of origin classification applying Hans’ algorithm. The graph shows 71 GCB (red line) and 96 NON-GCB (green line) DLBCL cases. A positivity threshold greater than 30% of tumor cells staining was employed for CD10, BCL6, and MUM1/IRF4 markers in order to calculate the Hans’ algorithm. D) Kaplan-Meier curves comparing EFS probabilities of 173 DLBCL patients according to Cell of origin classification applying Lymph2Cx (Nanostring). The graph shows 81 GCB (red line), 31 Unclassified (green line) and 61 ABC (purple line) DLBCL cases. E) Sankey plot showing the comparison of the assignment of each COO subgroup defined by applying the Hans algorithm and by using the Lymph2Cx assay on the Nanostring platform. F) Kaplan-Meier curves comparing EFS probabilities of 172 DLBCL patients according to BCL2 and MYC expressions. A positivity threshold of 40% was used for MYC immunohistochemistry and 50% for MYC immunohistochemistry. The assessment of double-expressor (DE) phenotype was established when both markers were positive. The graph shows 5 BCL2-/MYC+ (green line), 53 BCL2-/MYC- (yellow line), 94 BCL2+/MYC- (purple line) and 20 BCL2+/MYC+ (red line) DLBCL cases. The number of patients in each group is reported; P values refer to log-rank test.

**Figure S2. Sequencing statistics and RNA-Seq analysis.** A) Bar chart plot showing the RNA sequencing run statistics. Specifically, plots highlight the percentage of reads unmapped (red), multimapped (yellow) and uniquely mapped (blu) calculated on the total of reads obtained per case. B) Heatmap graph showing consistency between COO classification according to Lymph2Cx and RNA-Seq (0.97 Kappa Cohen). Clustering was calculated by evaluating the different expression levels of Lymph2Cx genes (15 genes), using RNA-Seq data, on 142 cases classified as ABC or GCB by Lymph2Cx. C) Differential pathway usage between GCB (blue bar) and ABC (red bar) cases. Left panel reports the heatmap of significant GSVA enrichment scores for ABC/GCB in DLBCL bulk RNA-Seq data. The right dotplot reports GSEA normalized enrichment scores scaled by -log_10_(FDR).

**Figure S3. EcoTyper analysis comparison.** A) Heatmap graph showing differently expressed genes utilized by EcoTyper for B cell state classification. On the left differently expressed genes were calculated on our DLBCL cohort (178 cases). Heatmap on the right shows a comparison with the Schmitz et al. cohort of DLBCL (438 cases), as reported by EcoTyper.

**Figure S4. Sankey diagram distributions.** Sankey plot showing the comparison of the assignment of each COO subgroup defined by applying the Lymph2Cx assay on the Nanostring platform with A) the B cell state as determined by EcoTyper and B) the Lymphoma Ecotypes as labeled by EcoTyper.

**Figure S5. Tumor microenvironment cell states associated survival curves.** Kaplan-Meier curves comparing EFS probabilities of our DLBCL cohort. Graphs are divided for the 12 tumor microenvironment (TME) cell types. Each Kaplan Meier indicates the cell state classification for a specific TME cell type. P values refer to log-rank test.

**Figure S6. Cell states co-occurrence analysis.** Co-occurrence patterns of cell states (B cells, and tumor microenvirnment cells) calculated using the Jaccard index adjusted for statistical significance.

**Figure S7. Lymphgen analysis.** A) Pie chart indicating molecular cluster classification according to Lymphgen algorithm on our cohort (146 DLBCL). Specifically, 22 BN2 (Blue), 16 EZB (light green), 18 MCD (orange), 3 N1 (dark green), 20 ST2 (purple), and 67 labeled as “Other” (light blue). B) Kaplan-Meier curves comparing EFS probabilities of 69 DLBCL patients divided by genetic features by applying the Lymphgen algorithm. The graphs shows 17 BN2 (blue line), 15 EZB (light green line), 16 MCD (orange line), 3 N1 (dark green line) and 18 ST2 (light purple line) DLBCL cases. The number of patients in each group is reported; P values refer to log-rank test. C) Sankey plot showing the comparison of the assignment of each B cell state subgroup defined by applying the EcoTyper algorithm, and the molecular clusters obtained from Lymphgen.

**Figure S8. Mutational characterization of B cell states.** A) Clustering of mutation percentages normalized by Z-score, enriched in A) the 5 different B cell states (S1, S2, S3, S4, S5) and B) in the 3 B cell states subgroup obtained by recursive partitioning (S1, S2-S3-S4, S5). Only genes mutated in more than 5% of patients were considered.

**Figure S9. Overall survival according to B cell states and Lymphoma Ecotypes classifications.** A) Kaplan-Meier curves comparing overall survival (OS) probabilities of 176 DLBCL patients according to B cell state classification. The graph shows 46 S1 (red line), 42 S2 (yellow line), 23 S3 (green line), 29 S4 (blue line) and 36 S5 (purple line) DLBCL cases. B) Kaplan-Meier curves comparing OS probabilities of 176 DLBCL patients grouped according to recursive partitioning of B cell state classification. The graph shows 46 S1 (red line), 94 S2-S3-S4 (green line) and 36 S5 (purple line) DLBCL cases. C) Kaplan-Meier curves comparing overall survival (OS) probabilities of 176 DLBCL patients according to Lymphoma Ecotypes (LE) classification. The graph shows 31 LE1 (blue line), 19 LE2 (light blue line), 5 LE3 (light purple line), 20 LE4 (dark green line), 12 LE5 (light green line), 17 LE6 (yellow line), 37 LE7 (orange line), 23 LE8 (ochre line) and 12 LE9 (red line) DLBCL cases. D) Kaplan-Meier curves comparing OS probabilities of 176 DLBCL patients grouped according to recursive partitioning of LE cell state classification. The graph shows 55 LE1-LE2-LE3 (purple line), 49 LE4-LE5-LE6 (green line), 60 LE7-LE8 (yellow line) and 12 LE9 (red line) DLBCL cases.

**Figure S10. Transcriptomic enrichment profiling of B cell state signatures in DLBCL cell lines.** A) Heatmap depicting row-wise Z-score-transformed Normalized Enrichment Scores (NES) obtained via ssGSEA across five human DLBCL cell lines (SU-DHL-2, SU-DHL-8, SU-DHL-10, OCI-Ly7, and RI-1) evaluated for the EcoTyper B cell states. B) ssGSEA enrichment heatmap of curated DLBCL-specific functional and oncogenic pathways across the profiled cell lines.
